# Diseño y validación de un cuestionario para medir acceso y calidad de los servicios de aborto en Argentina

**DOI:** 10.18294/sc.2025.5348

**Published:** 2025-04-22

**Authors:** Brianna Keefe-Oates, Mercedes Krause, Agustina Ramón Michel, Silvina Ramos, Mariana Romero

**Affiliations:** 1 PhD in Population Health Sciences. Investigadora posdoctorado, Roux Institute, Northeastern University, Maine, EEUU. bkeefeoates@gmail.com Northeastern University Roux Institute Northeastern University Maine USA bkeefeoates@gmail.com; 2 Doctora en Ciencias Sociales. Investigadora, Centro de Estudios de Estado y Sociedad, Instituto de Investigaciones Gino Germani, Universidad de Buenos Aires, Ciudad Autónoma de Buenos Aires, Argentina. merkrause@gmail.com Universidad de Buenos Aires Instituto de Investigaciones Gino Germani Universidad de Buenos Aires Ciudad Autónoma de Buenos Aires Argentina merkrause@gmail.com; 3 Magíster en Derecho. Investigadora Asociada, Centro de Estudios de Estado y Sociedad, Instituto de Investigaciones Gino Germani, Universidad de Buenos Aires, Ciudad Autónoma de Buenos Aires, Argentina. rmichelagus@gmail.com Universidad de Buenos Aires Centro de Estudios de Estado y Sociedad Instituto de Investigaciones Gino Germani Universidad de Buenos Aires Ciudad Autónoma de Buenos Aires Argentina rmichelagus@gmail.com; 4 Socióloga. Investigadora titular, Centro de Estudios de Estado y Sociedad, Instituto de Investigaciones Gino Germani, Universidad de Buenos Aires, Ciudad Autónoma de Buenos Aires, Argentina. silvinaramosarcoiris@gmail.com Universidad de Buenos Aires Centro de Estudios de Estado y Sociedad Instituto de Investigaciones Gino Germani Universidad de Buenos Aires Ciudad Autónoma de Buenos Aires Argentina silvinaramosarcoiris@gmail.com; 5 Magíster en Salud Reproductiva, Investigadora independiente, Consejo Nacional de Investigaciones Científicas y Técnicas. Investigadora titular, Centro de Estudios de Estado y Sociedad, Instituto de Investigaciones Gino Germani, Universidad de Buenos Aires, Ciudad Autónoma de Buenos Aires, Argentina. mromero@cedes.org Universidad de Buenos Aires Centro de Estudios de Estado y Sociedad Instituto de Investigaciones Gino Germani Universidad de Buenos Aires Ciudad Autónoma de Buenos Aires Argentina mromero@cedes.org

**Keywords:** Aborto, Salud Sexual, Salud Reproductiva, Encuestas y Cuestionarios, Monitoreo Sanitario, Argentina

## Abstract

En Argentina, la Ley 27610, que legalizó la interrupción voluntaria del embarazo, fue el resultado de años de activismo y alianzas políticas. Para monitorear su implementación, entre 2022 y 2024 se desarrolló un instrumento que evalúa la accesibilidad y calidad de los servicios de aborto, considerando la experiencia de las personas usuarias. Realizamos un estudio en tres etapas, incluyendo paneles de personas expertas, entrevistas cognitivas, y una prueba piloto del instrumento con personas que abortaron. Estos esfuerzos resultaron en el cuestionario Medimos Accesibilidad y Calidad en los Servicios del Aborto (MACA) que se puede aplicar en el sistema de salud en Argentina. Este cuestionario se puede usar para propósitos de monitoreo, para identificar mejoras en los servicios de salud y desigualdades en el acceso según territorios y características sociales.

## INTRODUCCIÓN

El 30 de diciembre de 2020, el Congreso de la Nación Argentina sancionó la Ley 27610 de Acceso a la Interrupción Voluntaria del Embarazo, cerrando así un largo proceso de movilización social y de espera política. Este hito legal vino acompañado del hecho sociológico que supuso la “marea verde”^1^, que se extendió como inspiración en América Latina y otras regiones. Desde entonces, y hasta diciembre de 2023, la puesta en marcha de una política pública nacional de acceso al aborto tuvo como pilares la distribución de insumos para la interrupción del embarazo a todas las provincias y la producción pública de misoprostol para garantizar su disponibilidad y accesibilidad económica^2^.

En 2023, mediante la Disposición 1470/2023, la Administración Nacional de Medicamentos, Alimentos y Tecnología Médica (ANMAT) autorizó el registro de la mifepristona, un medicamento que amplía las opciones terapéuticas y ofrece mayor seguridad y eficacia en el aborto con medicamentos, en sintonía con las recomendaciones internacionales de la Organización Mundial de la Salud (OMS)^3^. Además, la Dirección Nacional de Salud Sexual y Reproductiva, órgano rector dependiente del Ministerio de Salud de la Nación, elaboró más de 40 documentos para la orientación de la gestión y las prácticas clínicas. Estos fortalecieron la articulación con las provincias y un clima de trabajo seguro y previsible para los equipos de salud. También se llevaron adelante capacitaciones para la realización de la técnica de aspiración manual endouterina (AMEU) en las que participaron cerca de 500 profesionales entre 2021 y 2023, ampliando así los métodos disponibles para abortar. Como resultado, se duplicó la cantidad de los servicios públicos de salud que garantizan la interrupción voluntaria del embarazo y la interrupción legal del embarazo (IVE-ILE), pasando de 907 en 2020 a 1.982 en 2023, y se multiplicó por nueve la cantidad de tratamientos para el aborto con medicamentos distribuidos, pasando de 18.590 en 2020 a 166.164 en 2023^4^. 

Este proceso de institucionalización y puesta en práctica de la Ley de Acceso a la Interrupción Voluntaria del Embarazo en Argentina introdujo por primera vez, de manera masiva y en todo el sistema de salud, tecnologías reproductivas como el misoprostol y la mifepristona y protocolos de atención acordes a los estándares internacionales. Se trata de cambios históricos en la accesibilidad y calidad de la atención de los servicios de aborto, que requieren de seguimiento para asegurar que los derechos reconocidos sean garantizados en la vida de las mujeres y otras personas gestantes, y que los cambios producidos en los procesos de atención mejoren las experiencias de las personas usuarias que buscan realizar un aborto (referidas de aquí en adelante como “usuarias”). 

A diferencia de la accesibilidad, la calidad en la atención resulta una dimensión crítica de la política de aborto, que la OMS ha destacado recientemente, y sobre la cual no disponemos de indicadores estandarizados en Argentina^3^. Conocer esta dimensión y observar sus características en los servicios de salud es una deuda del monitoreo de la política de aborto que requiere de mediciones especiales. 

Los antecedentes en la literatura académica muestran una falta de acuerdo acerca de cuáles son los estándares para medir la calidad de los servicios de aborto. En una revisión de los indicadores utilizados entre 2008 y 2018 para medir los avances en la atención del aborto, Filippi *et al*. encontraron casi 800 indicadores, de los cuales solo el 22% se repite a través de las fuentes y, en general, corresponden a las dimensiones de prevalencia e incidencia del aborto^5^.

La calidad de la atención pocas veces se mide desde la perspectiva de las personas usuarias^6^. Algunas excepciones se pueden encontrar en la evaluación de Billings y Benson sobre América Latina^7^, estudios previos a la Ley 27610 sobre la calidad de la atención posaborto en hospitales públicos de Tucumán y Ciudad Autónoma de Buenos Aires^8^^,^^9^, y un estudio cualitativo sobre las preferencias y prioridades de quienes abortan respecto de sus interacciones con acompañantes y profesionales de salud en Buenos Aires y Neuquén^10^. Son más comunes los cuestionarios de calidad de la atención centrados en quienes proveen la atención del aborto o en los servicios de salud, relevando dimensiones estructurales como la infraestructura, los tiempos de espera, o las competencias técnicas para el manejo efectivo del dolor^5^^,^^10^^,^^11^. Cuando se tiene en cuenta a las personas usuarias, en general, se utilizan rankings de puntuación para medir la satisfacción^12^. Este tipo de instrumentos tiende a resultar en altos grados de satisfacción, incluso en contextos en los que la calidad de la atención es baja, y por ello se ha cuestionado si el grado de satisfacción es un indicador válido para medir el nivel de calidad de la atención del aborto^13^^,^^14^.

En este contexto, en 2018 las organizaciones internacionales Metrics for Management (M4M), Ibis Reproductive Health e Ipas colaboraron para liderar la Iniciativa para la Calidad de los Servicios de Aborto (*Abortion Service Quality Initiative,* ASQ por sus siglas en inglés) y desarrollaron el primer estándar global, la Herramienta de Calidad de la Atención al Aborto (ACQTool, por sus siglas en inglés) que mide la calidad de los servicios de aborto en países de ingresos bajos y medios^12^^,^^15^.

En sintonía con estos esfuerzos, desde el “proyecto mirar”, una iniciativa del Centro de Estudios de Estado y Sociedad (CEDES) e Ibis Reproductive Health, que monitorea la implementación de la Ley de Interrupción Voluntaria del Embarazo en Argentina, desarrollamos, en los últimos tres años, el cuestionario Medimos Acceso y Calidad del Aborto (MACA), con el propósito de desarrollar un instrumento para medir la accesibilidad y calidad de los servicios de aborto a ser usado ampliamente en el sistema de salud. El estudio tuvo como objetivo diseñar y validar un cuestionario anónimo y autoadministrado para evaluar la accesibilidad y la calidad de la atención luego de la legalización. En línea con el ACQTool y en consulta con una variedad de actores clave y partes interesadas en Argentina, el estudio MACA se propuso diseñar y validar un instrumento útil para nuestro contexto. En otras palabras, el cuestionario MACA es un instrumento estandarizado para medir el acceso y la calidad de los servicios de aborto desde la perspectiva de las personas usuarias, específico para el contexto argentino y la Ley de Interrupción Voluntaria del Embarazo vigente. 

Al igual que la herramienta ACQTool, el cuestionario MACA mide la calidad de la atención desde la perspectiva de las personas usuarias, abordando sus experiencias subjetivas, los procesos y sus percepciones sobre la atención recibida. Tener en cuenta estos aspectos resulta fundamental para conocer la variabilidad de experiencias concretas al acceder y utilizar servicios de aborto. Las percepciones de las personas sobre la atención pueden influir en las creencias sobre el aborto, así como afectar la confianza de la población en el sistema de salud.

El objetivo de este artículo es describir el proceso de desarrollo del cuestionario MACA, instrumento estandarizado para medir el acceso y la calidad de los servicios de aborto desde la perspectiva de las personas usuarias, específico para el contexto argentino y la legislación sobre aborto vigente. Buscamos hacer explícitas las decisiones y los procedimientos para alcanzar la validez y confiabilidad del instrumento, definidas respectivamente como el grado en que el instrumento logra medir lo que se busca medir y si lo hace de manera consistente. En particular, mostramos evidencia en torno a tres tipos de validez: 1) la validez de contenido, es decir, el grado en que el instrumento refleja la accesibilidad y la calidad de los servicios, según los dominios que contienen estos dos conceptos en la literatura académica; 2) la validez de las personas expertas, es decir, el grado en que el instrumento refleja el constructo según las personas expertas en el tema; y 3) la validez asociada a la comprensión y aceptabilidad del instrumento por parte de las personas usuarias.

## METODOLOGÍA

**Figura 1:**
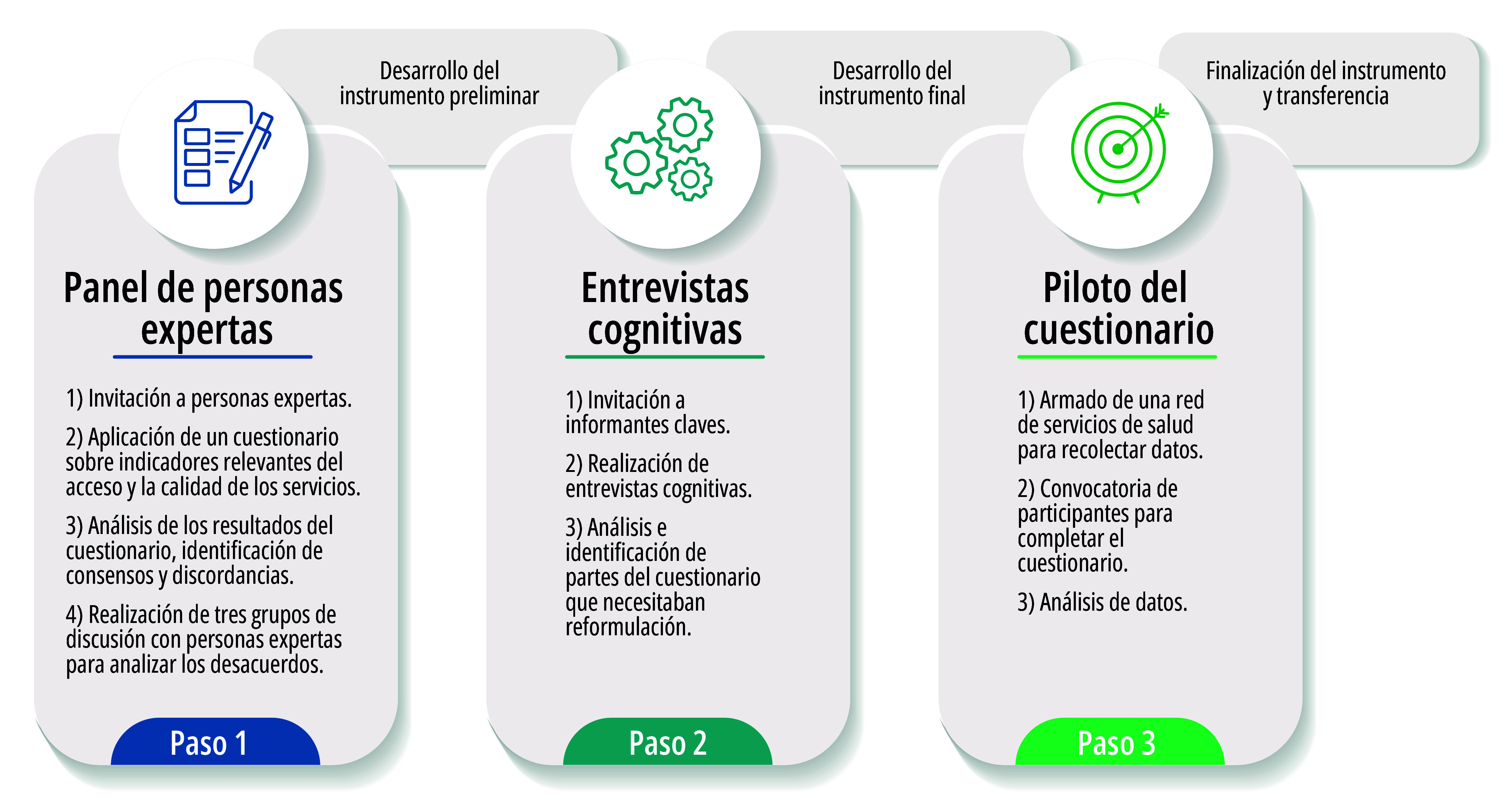
Pasos de la construcción del cuestionario Medimos Acceso y Calidad del Aborto (MACA). Argentina, 2023-2024.

Se trata de un estudio instrumental con métodos mixtos en tres etapas, para desarrollar un cuestionario. Estas incluyeron: 1) paneles de personas expertas para identificar indicadores prioritarios sobre acceso y calidad (lo que incluyó tanto cuestionarios como grupos de discusión); 2) entrevistas cognitivas con personas expertas para obtener retroalimentación sobre el instrumento; y 3) prueba piloto del instrumento cuantitativo con personas que abortaron (Figura 1).

Fuente: Elaboración propia.

### Etapa 1: Paneles de personas expertas

El objetivo fue identificar una lista de indicadores relevantes y necesarios para medir el acceso y la calidad del aborto en el contexto argentino. Adaptamos la técnica de grupo nominal y el método Delphi para construir la lista de indicadores que el instrumento mediría con el fin de identificar barreras y brechas en el acceso y la calidad de los servicios de aborto desde la perspectiva de las personas que los utilizan para acceder a un aborto^16^^,^^17^. Con los paneles de las personas expertas, reunimos las opiniones y los conocimientos de una variedad de personas para arribar a un consenso acerca de cuáles de los indicadores propuestos eran los más relevantes para medir cada una de las dimensiones de accesibilidad y calidad de los servicios de aborto. De esta forma, buscamos llegar a la “validez de las personas expertas”^18^.

Partimos de 42 indicadores con el fin de reducir esta lista a aquellos indicadores relevantes y prioritarios para el contexto argentino. Recurrimos a los indicadores de calidad de los servicios de aborto estandarizados por ACQTool y a la literatura sobre accesibilidad y calidad de los servicios de salud en general y de aborto en particular^24^. Siendo un equipo bilingüe, con investigadoras argentinas y estadounidenses, con expertise en salud sexual y (no) reproductiva, tradujimos los indicadores del inglés al castellano y adaptamos, en parte, las propuestas internacionales. Después, a través de los paneles de personas expertas, se revisaron estos indicadores para asegurar una gramática correcta y adecuada al contexto local. También revisamos el sistema de información del “proyecto mirar” que, previo al diseño del cuestionario MACA, había identificado áreas de vacancia en las fuentes secundarias disponibles para medir las acciones y los cambios en la implementación de la política pública de acceso al aborto. Este proceso de selección estratégica de indicadores en el sistema de información para el monitoreo del “proyecto mirar” se basó en dos reconocidos marcos de evaluación para relevar y definir conceptualmente indicadores específicos, medibles y factibles: el Monitoreo de Resultados para la Equidad (MoRES) y el de Availability, Accessibility, Acceptability, Quality (AAAQ)^25^^,^^26^^,^^27^. Esta lista inicial de indicadores para el instrumento del cuestionario MACA con la que comenzó el proceso de consulta a las personas expertas incluía indicadores que correspondían a cinco dimensiones: una de acceso y cuatro de calidad. Las dimensiones de calidad, adaptadas de las dimensiones identificadas en el ACQTool, incluyeron: apoyo/acompañamiento/contención, provisión de información, toma de decisiones, y competencia técnica. 

Los paneles de personas expertas se organizaron en dos fases: un cuestionario sobre sus valoraciones acerca de los indicadores propuestos y tres grupos de discusión acerca de los indicadores sobre los que no había acuerdo según los resultados del cuestionario. 

Convocamos a una muestra intencional para participar en el panel. Las personas participantes incluyeron tres perfiles de profesionales involucrados en la gestión y provisión de servicios de aborto: a) proveedores de servicios de aborto; b) líderes de organizaciones de mujeres; y c) tomadores de decisión vinculades a la implementación de la Ley 27610. Todas las personas expertas tenían, por lo menos, cinco años de trabajo en el campo de servicios de aborto. 

En el grupo de proveedores de servicios, buscamos representar tanto al sector de salud pública (abarcando la provisión de servicios en los niveles de atención primaria, secundaria y terciaria), al sector privado y al sector comunitario (acompañantes de personas que autogestionan su aborto fuera del sistema de salud). En el grupo de organizaciones de mujeres, buscamos incluir a organizaciones de incidencia territorial y acompañamiento en la realización de abortos. En el grupo de tomadores de decisión, invitamos a personas vinculadas a la gestión de políticas, programas y servicios a nivel nacional, provincial y municipal. Además, nos esforzamos por asegurar la diversidad geográfica en cada grupo, conformando así tres grupos de entre 10 y 11 participantes.

Inicialmente, administramos un cuestionario, a través de la plataforma Qualtrics, a las personas expertas que consintieron en participar. Este cuestionario incluía una lista de indicadores previamente seleccionados por el equipo de investigación. Los participantes debían evaluar el grado de relevancia de cada indicador para medir la accesibilidad o calidad, asignando un valor del 1 (que representaba “No relevante”) al 5 (que representaba “Muy relevante”).

Una vez completados los cuestionarios, desarrollamos un sistema para identificar indicadores con una concordancia mayoritaria para su inclusión o eliminación (es decir; indicadores con puntajes que reflejaban un alto grado de acuerdo entre las personas expertas) y aquellos en los que no había concordancia. 

Seleccionamos los indicadores para los grupos de discusión siguiendo los siguientes pasos y criterios:

1. Identificamos los indicadores cuyas puntuaciones tenían alta concordancia y se incluirían directamente en el instrumento, sin necesidad de ser tratados en los grupos de discusión porque: 1) todas las respuestas los puntuaban entre 3 a 5 (desde neutral hasta “muy relevante”, o 2) al menos el 92% de las personas expertas los habían evaluado con un 4 (relevante) o 5 (muy relevante). También incluimos indicadores del ACQTool que consideramos que no se duplicaban con ningún otro indicador.
2. Decidimos descartar los indicadores con menos apoyo entre les expertes: menos del 80% de les expertes determinaron que el indicador fue “relevante” o “muy relevante” o entre el 80 y 91% de les expertes eligieron “relevante’ o ‘muy relevante’ pero el promedio de todas las respuestas juntas estaba en el tercil más bajo de respuestas, porque un grupo de encuestades lo evaluaron como no relevante.
3. Los indicadores restantes (es decir, aquellos en los que no hubo concordancia clara sobre su inclusión o exclusión en el instrumento) fueron debatidos durante los grupos focales.

Reunidos estos datos cuantitativos, procedimos a compartir por correo electrónico un informe de resultados a las personas expertas, identificando las concordancias y discordancias en las respuestas. Después, convocamos a los tres grupos focales de discusión de manera virtual, uno con cada subgrupo de expertos, conformados por quienes habían respondido el cuestionario previamente. De ellas, un total de 27 personas participaron en los grupos focales; entre 9 y 11 personas en cada discusión. 

Abrimos los grupos con una breve presentación de los resultados, para asegurarnos de que todas las personas contaran con la información necesaria, y después las guiamos en una discusión abierta centrada en cada uno de los indicadores sobre los que no había concordancia según los resultados del cuestionario. Ordenados temáticamente, estos indicadores se mostraron en pantalla junto a los indicadores que habían sido incluidos directamente y se solicitó a las personas participantes que reflexionaran acerca de su contenido: ¿ya es abordado por otro indicador?; ¿por qué sería necesario incluir este indicador en el instrumento final? Al final de la discusión de cada indicador, buscamos arribar a un consenso sobre la utilización o no de este indicador basado en los argumentos expuestos. En algunos casos, el grupo de discusión sugirió modificaciones o un nuevo indicador, lo que fue llevado como sugerencia al próximo grupo para discutirlo de forma iterativa.

Estos grupos se realizaron por la plataforma Zoom y fueron grabados, previo consentimiento. Todas las personas participantes recibieron un vale de compra en una librería como reconocimiento por su participación. Dos investigadoras revisaron las grabaciones para documentar las opiniones de cada indicador que surgieron en cada grupo. El equipo de investigación en conjunto revisó estas sugerencias. En los casos de discordancias entre los grupos, incorporamos la sugerencia cuando dos de los tres grupos acordaban, o según nuestra propia expertise, cuando las sugerencias y modificaciones no podían refrendarse con los grupos anteriores o bien cuando reconocimos que este indicador era relevante y no podría recolectarse de otra fuente de datos. 

### Etapa 2: Entrevistas a informantes calificadas

Después de determinar los indicadores para el instrumento, el equipo de investigación desarrolló una versión preliminar que contenía preguntas correspondientes a los indicadores elegidos. Para cada indicador de la iniciativa Abortion Service Quality (ASQ), utilizamos la pregunta correspondiente, revisando el lenguaje para adaptarlo al contexto local. Para indicadores sin preguntas definidas por ASQ, adaptamos preguntas de estudios previos y/o formulamos la pregunta. 

El instrumento contenía 54 preguntas. Incluía 17 preguntas sobre la persona y el aborto realizado para captar diferencias en la accesibilidad y calidad según distintas características sociodemográficas y experiencias (por ejemplo, según subsector en el que se atendió, edad gestacional). También incluía 11 preguntas que correspondían a los indicadores de acceso y 26 que correspondían a los indicadores de calidad determinados en los paneles de personas expertas.

Una vez estructurado el instrumento, buscamos validar el contenido y la redacción de las preguntas. Utilizando la técnica de entrevistas cognitivas para validación del contenido de un cuestionario, hicimos entrevistas a informantes calificadas, ya que por consideraciones logísticas y razones éticas no se pudo hacer entrevistas cognitivas a personas que hubiesen abortado recientemente^19^. Las entrevistas se realizaron a integrantes de organizaciones de mujeres que apoyan a personas que buscan servicios de aborto, proveedoras de servicios de aborto y académicas con al menos dos años de experiencia en el tema. Convocamos a una muestra intencional, basada en nuestro conocimiento sobre el campo, identificando a personas que representaban una diversidad de experiencias en servicios de aborto de diferentes provincias. Ninguna de estas personas había participado de los grupos focales. 

Las nueve entrevistadas fueron convocadas por correo electrónico, y si aceptaban, recibían el borrador del instrumento para ofrecerles la oportunidad de revisarlo de forma previa al encuentro. Las entrevistas se llevaron a cabo por Zoom. Después del proceso de consentimiento informado solicitamos a las entrevistadas que asumieran el lugar de una usuaria, leyeran las preguntas del cuestionario y pensaran cómo las responderían luego de un aborto. En las distintas secciones les solicitamos que identificaran las preguntas o categorías de respuesta que resultaran confusas y que sugirieran alternativas. También, indagamos por un subgrupo de preguntas y categorías de respuestas para confirmar sus interpretaciones con mayor profundidad. Al final, preguntamos sobre sus preferencias de vocabulario, incluyendo el uso de “IVE/ILE” en vez de “aborto”, cómo referirse a las personas que contestarían el cuestionario (por ejemplo, pacientes, usuarias, personas que solicitan abortos), y la preferencia sobre el verbo ligado al aborto como “realizar”, “obtener”, o “tener” un aborto. Cada participante recibió una retribución monetaria en reconocimiento del tiempo dedicado.

Las entrevistas fueron grabadas y se tomaron notas. Sobre esta base, se confeccionó una tabla con los cambios sugeridos por cada participante. Luego, el equipo completo revisó las sugerencias y modificó el instrumento según las sugerencias.

### Etapa 3: Piloto del instrumento

Se realizó una prueba piloto del instrumento en formato de cuestionario con personas que habían accedido a un aborto en servicios de salud del país con los siguientes objetivos:

1. Evaluar la factibilidad de autoadministrar el instrumento y los procedimientos necesarios para hacerlo.
2. Validar las preguntas del instrumento y determinar necesidades de revisión.
3. Estandarizar los procedimientos logísticos para la administración del instrumento.
4. Analizar los datos preliminares para entender la calidad y el acceso en los servicios donde se realizó la prueba piloto del instrumento.

El piloto se realizó en 11 servicios de salud entre junio 2023 y abril 2024 (Tabla 1) elegidos intencionalmente para representar una variedad de contextos geográficos y del sistema de salud, así como experiencias según métodos de aborto y tipo de establecimiento. Elaboramos un manual para el trabajo de campo y capacitamos, a profesionales de la salud que proveían abortos, en el contenido del cuestionario los procedimientos para identificar personas usuarias elegibles y hacer la recolección de datos.

**Tabla 1:** Establecimientos participantes de la prueba piloto del cuestionario Medimos Acceso y Calidad del Aborto (MACA). Argentina, 2023-2024.

Características	Número de servicios (n=11)
**Provincia del establecimiento**	
Jujuy	4
Santa Fe	3
Buenos Aires	1
Entre Ríos	1
Salta	1
Neuquén	1
**Tipo de establecimiento**	
Hospital	5
Centro de atención primaria	4
Clínica	2
**Subsector de salud**	
Público	9
Privado	1
Obras sociales	1
**Método de aborto ofrecido**	
Medicamentos y AMEU	5
Solo medicamentos	4
Solo AMEU	2

Fuente: Elaboración propia. AMEU= aspiración manual endouterina.

Para ser elegibles, las personas tenían que tener al menos 16 años de edad, haberse realizado un aborto en los últimos tres meses en las instituciones seleccionadas para el piloto, poder hablar y leer castellano, y no haber realizado el aborto por su cuenta o solo con acompañamiento de una grupa feminista o comunitaria sin haber recibido atención previamente en el servicio. Una vez completado el aborto y tras el alta médica, las y los profesionales de salud en cada institución, que recibieron capacitación en el estudio, invitaron a las personas usuarias a participar de la encuesta. Como parte del proceso de invitación, cada profesional compartió información sobre los objetivos, procedimientos y aspectos relativos al anonimato y confidencialidad del estudio. A cada persona interesada se le ofreció una hoja informativa sobre la investigación con un código QR para leer con su teléfono y el enlace al cuestionario en línea diseñado con el programa encriptado Qualtrics. Al escanear el código, se desplegaban preguntas que confirmaban el consentimiento informado y la elegibilidad para participar y luego se accedía a la encuesta.

La encuesta incluía preguntas sociodemográficas y preguntas que correspondían a los indicadores que se habían identificado a través de los paneles de personas expertas y se habían verificado en las entrevistas cognitivas. Su llenado implicaba de 15 a 20 minutos. 

Realizamos un análisis descriptivo de las características de la muestra y de las preguntas de acceso y calidad, con el objetivo de explorar la validez del instrumento, así como para identificar patrones de preguntas sin respuesta. Como las preguntas acerca de la calidad se enfocaban principalmente en percepciones, y estaban dentro de cuatro dimensiones, analizamos qué tanto estas preguntas reflejaban los distintos temas que debía medir el instrumento. Realizamos un análisis estadístico para medir las frecuencias de las respuestas y observar si había suficiente diferencia entre las respuestas para medir diferencias en la calidad. Después, medimos las correlaciones entre las respuestas sobre calidad para determinar si: 1) las preguntas que temáticamente eran opuestas se veían así en las correlaciones, y 2) si había preguntas con alta correlación que parecían reflejar un mismo tema y, por lo tanto, no era necesario que las dos fueran incluidas en el instrumento. También solicitamos retroalimentación de profesionales de salud quienes participaron en la recolección de datos, para entender la factibilidad de administrar el cuestionario en el campo y ajustar los procedimientos de acuerdo a esta retroalimentación. A partir de este análisis se hicieron cambios para arribar a una versión final.

El proyecto de investigación contó con la aprobación de los comités de ética y/o autoridades de cada una de las instituciones participantes. A su vez fue evaluado y aprobado por el comité de ética en investigación “Iniciativa y Reflexión Bioética”, perteneciente a Respire Centro Médico, y los siguientes comités provinciales: Comité Provincial de Ética de Investigación en Salud del Gobierno de Jujuy (Ref. expte.: 773-1251/2023); Comité de Ética en Investigación de la Secretaría de Salud Pública de la Municipalidad de Rosario; y la Comisión Provincial de Investigaciones Biomédicas del Ministerio de Salud Pública de Salta (Ref. expte.: 244-160192/2023-0 y Code. 1). No se incluye la información de cada uno de los comités y avales institucionales para resguardar el anonimato de los servicios en los que se recolectaron datos. Todos los datos fueron anonimizados y almacenados en archivos protegidos.

## RESULTADOS

### Etapa 1: Paneles de expertes

#### Primera fase: Encuesta a personas expertas

Se seleccionaron 35 personas expertas según los criterios indicados. Utilizando el análisis descrito más arriba, arribamos a 17 indicadores que incluimos directamente como indicadores finales para medir el acceso y la calidad, 13 para discutir en los grupos focales, y 12 que se excluyeron según los resultados de la encuesta y no merecían discusión. En general se observó que los indicadores elegidos a través de la encuesta con expertes ayudaron a enfocar el instrumento en temas directamente relacionados con el aborto, a la vez que dejaron por fuera otros con una relación más lejana, como los relacionados a servicios anticonceptivos. Tanto las medidas objetivas como subjetivas resultaron relevantes según les expertes. Por ejemplo, se solicitó la cantidad de días transcurridos desde que solicitó el aborto hasta que lo realizó, pero también la aceptabilidad de ese tiempo transcurrido para la persona usuaria (más de lo que hubiera esperado, lo que esperaba, menos de lo que hubiera esperado). 

Cuando se analizaron los resultados de la encuesta sobre indicadores de acceso hubo consenso en no incluir dos indicadores sobre gastos de bolsillo: “El costo de traslado hacia la/el proveedor es accesible para la usuaria” y “La usuaria pagó de su bolsillo por el servicio doméstico y/o de cuidados durante su proceso de aborto”, a la vez que se valoraron -aunque no hubo un consenso absoluto- otros directamente asociados con el gasto del procedimiento de aborto: “La usuaria pagó de bolsillo la ecografía para realizar el aborto” y “La usuaria pagó de bolsillo la medicación para realizar el aborto”. Estos dos indicadores fueron tratados en los grupos de discusion. 

Del análisis de los resultados de las preguntas enfocadas en indicadores de calidad, se descartaron dos indicadores de la dimensión “Apoyo/contención/acompañamiento”. Uno de estos indicadores, “Las usuarias se sintieron cómodas compartiendo información personal con el personal” se descartó por ser similar a “Las usuarias confiaron en que la/el proveedor mantendría la confidencialidad de su información personal”. Este último recibió más apoyo. “Las/los proveedores derivan y brindan información completa a las usuarias sobre otros servicios relevantes, según sea necesario (por ejemplo, servicios de salud sexual y reproductiva, servicios legales, violencia de género, etc.)” se excluyó porque se enfocaba en temas más amplios que el aborto. También, en la dimensión de “Provisión de información” se descartó el siguiente indicador: “Las usuarias sabían qué esperar de cada paso de su visita/provisión de atención”, por ser similar a otros indicadores en esta dimensión. 

#### Segunda fase: Grupos de discusión

En los grupos de discusión las personas participantes se enfocaron en incluir o no algunos indicadores y, en algunos casos, sugirieron añadir nuevos indicadores. También hicieron sugerencias sobre el lenguaje. En general, acordaron descartar indicadores referidos a expectativas y dejar los referidos a la percepción de las acciones del equipo de salud. 

Los grupos también sugirieron indicadores que no habían sido considerados o que, por los resultados del cuestionario, se habían quitado de la lista. Por ejemplo, aunque los resultados del cuestionario señalaron menos interés en medir la accesibilidad geográfica a través de un indicador sobre traslado, el tema fue mencionado varias veces en los grupos de discusión y se decidió añadir de nuevo un indicador sobre las barreras geográficas, medido en tiempo de traslado hacia los servicios de aborto visitados, como medida de accesibilidad. También se sugirió un indicador más global de gastos de bolsillo, que aparecía como un tema importante para medir la accesibilidad según les participantes. Finalmente, otro tema discutido fue la importancia de recibir información sobre todos los métodos disponibles para abortar, un indicador que no estaba previsto.

Todos los grupos realizaron distinciones entre apoyo, acompañamiento y alivio de las personas usuarias que buscan un aborto. En el marco de ese intercambio, surgieron recomendaciones respecto de relevarlos por separado porque podrían reflejar aspectos diferentes de la calidad. Mientras que algunas personas consideraron que el apoyo y acompañamiento estaban relacionados con la contención recibida durante el proceso de atención, otras interpretaron el apoyo como algo que se recibe en un momento de la consulta, a la vez que el acompañamiento podría entenderse como la presencia continua durante todo el proceso del aborto. El alivio, según las personas participantes, se asocia a lograr el aborto; las personas usuarias pueden sentir alivio al lograr “resolver” el aborto, pero sin un acompañamiento apropiado. Al final de estas discusiones, y al evaluar los otros indicadores para asegurar que no hubiera duplicación, se optó por un nuevo indicador: “Las personas usuarias se sintieron acompañadas a lo largo de su proceso de aborto”, el cual pareció más apropiado para medir el apoyo y el acompañamiento en todo momento, mientras que otros indicadores medían otros tipos de apoyo (por ejemplo, respeto) en el momento de la atención en el servicio de salud. 

Finalmente, durante el grupo de discusión, hubo sugerencias para lograr mayor precisión en los datos sociodemográficos a relevar y términos a usar en el cuestionario. Por ejemplo, en dos grupos de discusión se insistió en relevar el subsector de salud en el que se recibió atención, para medir la calidad por subsector. En otro grupo se sugirió una redacción específica en los indicadores: usar el término “personas usuarias” para asegurar un lenguaje inclusivo y, al preguntar sobre experiencias, incluir “a lo largo de todo el proceso de aborto”, para no referir a un solo momento. 

#### Tercera fase: Elección final de indicadores

En muchos casos hubo acuerdo entre los tres grupos para incluir o excluir un indicador. Los desacuerdos entre los grupos se dieron en torno a la redacción de un indicador y no de los temas. En la mayoría de los casos, las investigadoras incorporamos las sugerencias sobre la redacción. En los casos en que no hubo concordancia entre los grupos, elegimos el indicador seleccionado por dos de los tres grupos. Arribamos a 25 indicadores (Tabla 2) en las cinco dimensiones agrupadas en acceso y calidad, y después desarrollamos el instrumento sobre la base de estos indicadores. 

**Tabla 2:** Indicadores finales para medir el acceso y la calidad de los servicios de aborto en Argentina. Cuestionario Medimos Acceso y Calidad del Aborto (MACA). Argentina, 2023-2024.

Categoría	Dimensión	Indicador final	Fuente
Acceso	Acceso	La persona usuaria sabía dónde ir para solicitar un aborto	Desarrollado por el equipo de investigación
		Cantidad de dí­as transcurridos entre que solicitó el aborto por primera vez hasta que obtuvo el aborto o le dieron la medicación para el aborto	ACQTool
		El tiempo de espera desde el momento en que solicitó por primera vez un servicio de aborto hasta que obtuvo el aborto o le dieron la medicación para el aborto es aceptable para las personas usuarias	ACQTool
		La persona usuaria sabía que podía solicitar un aborto en un servicio de salud público, privado y de obras sociales	Desarrollado por el equipo de investigación
		La persona usuaria tuvo algún/os gasto/s directo de bolsillo vinculado al aborto (ecografía, medicación)	Desarrollado en el proceso de los grupos de discusión
		Cantidad de veces que tuvo que ir a un servicio de salud hasta que pudo realizarse el aborto	Desarrollado en el proceso de los grupos de discusión
		Tiempo total que tuvo que viajar para realizarse el aborto desde que lo solicitó hasta que lo obtuvo (microtiempos para resolver el aborto, incluye esperas para transporte)	Desarrollado en el proceso de los grupos de discusión
Calidad	Apoyo	Las personas usuarias recibieron información sobre el proceso de aborto acorde a sus expectativas	Sistema de información del proyecto mirar
		La persona usuaria se sintió respetada en todo momento	ACQTool
		Las personas usuarias confiaron en que la/el proveedor(es) mantendría(n) la confidencialidad de su información personal	ACQTool
		Las personas usuarias se sintieron acompañadas a lo largo de su proceso de aborto	Desarrollado en el proceso de los grupos de discusión
	Información	La/el proveedor verificó la comprensión de la información recibida por parte de las personas usuarias	ACQTool
		Las personas usuarias sabían qué hacer si experimentaban señales de alerta o en caso de complicaciones	ACQTool
		Las personas usuarias sabían cómo determinar si su aborto estaba completo	ACQTool
		Entre las personas usuarias que desean información sobre anticoncepción: La/el proveedor brinda información completa sobre anticoncepción	ACQTool
		La persona usuaria recibió toda la información sobre lo que iba a pasar durante su aborto	ACQTool
		La persona usuaria se sintió cómoda para expresar sus necesidades, preguntas y temores a el/la/los/las prestador(es)	ACQTool
		La persona usuaria recibió y entendió la información sobre la seguridad de todos los métodos de aborto disponibles	ACQTool
		La persona usuaria recibió información sobre la variedad de métodos disponibles para abortar	Desarrollado en el proceso de los grupos de discusión
	Decisiones	La persona usuaria sintió que el/la/lxs proveedor(es) apoyo(aron) su decisión de abortar	ACQTool
		La usuaria sintió que el/la/lxs proveedor(es) no la presionaron ni para abortar ni para no abortar	Desarrollado en el proceso de los grupos de discusión
		Las personas usuarias recibieron el método del aborto que quería.	ACQTool
		La usuaria recibió el método anticonceptivo que quería luego del aborto	ACQTool, Sistema de información del proyecto mirar
	Competencia técnica	La/el proveedor le transmitió seguridad a la usuaria durante el procedimiento de aborto	ACQTool
		Las usuarias sintieron que su dolor se manejó de manera efectiva	ACQTool

Fuente: Elaboración propia.

### Etapa 2: Desarrollo del instrumento y entrevistas cognitivas

Para asegurar que el lenguaje y formato del instrumento fueran comprensibles, realizamos nueve entrevistas cognitivas entre septiembre y octubre de 2022. Las participantes realizaron sugerencias sobre la redacción y el formato del instrumento. En algunos casos, también sugirieron otras preguntas. Solo cambiamos aquellas que correspondían a los indicadores ya elegidos por los paneles de personas expertas, dado que había sido un proceso previo riguroso. Un cambio importante fue la decisión de usar el evocativo *usted* en lugar de *vos*, con la intención de que el cuestionario tuviera mayor aceptación en la población de todas las provincias. Se incorporaron las siguientes sugerencias específicas:

#### Aclaraciones

- Asegurar que sea muy claro sobre cuál servicio estamos preguntando, ya que puede ser que visitaran distintos servicios (y el instrumento no indaga sobre esos otros servicios). Se agregó la siguiente consigna al inicio de la sección “sobre su experiencia con quienes la/le atendieron durante el proceso de aborto”: “*Cuando nos referimos a ‘proceso de aborto’ queremos decir desde que consultó por primera vez en el lugar donde realizó el aborto hasta que lo resolvió. Por favor no incluya sus experiencias con otros profesionales o servicios de salud adonde fue o llamó, pero no accedió al aborto. Si recibió atención en más de un servicio, por favor responder sobre el servicio que le invitó a participar de la encuesta*”.
- Añadir opciones de respuesta sobre los gastos de bolsillo: mensajería y análisis de sangre, separar AMEU y legrado (en lugar de “procedimiento quirúrgico”).
- Aclarar muy específicamente el plazo de las preguntas (por ejemplo, “durante el proceso de aborto”, “en las consultas sobre el aborto”), y definir qué es “todo el proceso”.

#### Formulación de preguntas y formato

- No utilizar grillas en la encuesta. La grilla sobre la cantidad de información recibida sobre diferentes temas (cómo usar los medicamentos, qué iba a suceder durante el procedimiento, etc.) se transformó en un bloque de preguntas singulares.
- Desagregar las categorías de respuesta sobre el método de aborto para incluir la AMEU y el legrado por separado (en vez de “método quirúrgico”)

#### Sumar la opción de respuesta “No estoy segura/e”

- Confirmaron el uso de “realizar” el aborto (por ejemplo, “cuántas semanas de embarazo tenía al realizar el aborto”, “la seguridad de los métodos para realizar el aborto”).
- Vieron más favorable la palabra aborto que IVE/ILE, que es más formal y menos accesible.
- Confirmaron la utilización de “personas usuarias” y no “pacientes” o “adolescentes, mujeres y personas con capacidad de gestar”.
- Utilizar el pronombre personal “usted” para dirigirse a las personas usuarias y hacer el instrumento válido en todo el territorio argentino.

### Etapa 3: Piloto del instrumento

#### Proceso de convocatoria

Durante julio y octubre de 2023 y febrero y abril de 2024, se invitó a participar de la encuesta a personas que habían accedido a un aborto, en los últimos tres meses, en los servicios seleccionados. Invitamos a un total de 506 personas, de las cuales 254 respondieron a la invitación y tenían criterios de elegibilidad. Finalmente, 225 personas completaron total o parcialmente el cuestionario. Estos resultados implican una tasa de respuesta -correpondiente a quienes respondieron las preguntas y tenian criterios de elegibilidad- del 44,5%.

La mayoría de las personas que participaron residía en Jujuy (36,8%), Santa Fe (18,2%), provincia de Buenos Aires (16,4%) y Entre Ríos (9,8%). Las edades variaron, pero la mayoría tenía 26 años o más (58,6%). Un 51,1% tenía un nivel de educación de terciario o más, y un 30,2% secundario completo. La mayoría tenía hijos (55,6%), y un 58,2% solo tenía una cobertura de salud pública, de las cuales un 31,6% tenían obra social, y el 5,3% prepaga. El 42,2% de las personas accedió a un aborto con AMEU, el 48,0% con medicamentos y el 6,7% con medicamentos y AMEU. La gran mayoría (84,6%) de las personas encuestadas tenía menos de 12 semanas de gestación al momento de abortar. 

#### Consistencia del instrumento

Utilizamos dos indicadores para analizar la factibilidad del instrumento: 1) porcentaje de personas que completó el cuestionario, y 2) variaciones en la falta de respuesta según algunas características (como provincia, servicio que la invitó, y nivel educativo). El 92,8% de las personas completó el cuestionario en su totalidad. En los cuestionarios incompletos, analizamos dónde dejaron de responder porque podría haber representado un problema con el instrumento. Observamos que ocho personas abandonaron el cuestionario en la primera sección, sin ningún otro patrón posterior de no respuesta. También analizamos preguntas específicas que las personas encuestadas no hubieran respondido. Solo detectamos un lugar en el instrumento donde más del 5,9% no contestó las preguntas. En este caso, eran preguntas contenidas en una grilla donde la persona tenía que marcar dentro de ella las respuestas. Este diseño puede haber causado confusión por lo que en el instrumento final cambiamos las preguntas para que cada una se respondiera por separado. 

También analizamos las variaciones en las respuestas y las correlaciones entre preguntas para determinar si las preguntas midieron temas distintos y si a la vez se correlacionaron de la manera que se esperaba. Las preguntas vinculadas a la calidad variaron en sus opciones de respuestas, según la pregunta, y en general incluyeron tres opciones de respuesta que se incorporaron en el análisis de variaciones y correlaciones, y una opción para “no estoy segura/e” que no se incorporó en el análisis ya que consideramos que no aportaba información a la pregunta. Por ejemplo, en las preguntas sobre las percepciones de las personas profesionales de salud y su trato, las opciones incluyeron las siguientes respuestas: “Sí, en todo momento”, “Sólo en algunos momentos/frente algunas personas”, o “No, en ningún momento/ frente a nadie”. Hubo también una opción para decir “no estoy segura/e” que no se incluyó en el análisis de correlaciones. En la Tabla 3, presentamos el porcentaje que respondió, “Sí, en todo momento”, u otra respuesta muy afirmativa, para señalar de forma sencilla las tendencias de las respuestas. Las variaciones entre respuestas dependían de la pregunta en sí: mientras hubo algunas con porcentajes muy altos de respuestas afirmativas, hubo otras preguntas con mayor variabilidad en las respuestas. La Tabla 3 muestra las preguntas que tuvieron las respuestas positivas más altas y bajas. 

**Tabla 3:** Respuestas positivas sobre las preguntas referidas a calidad. Cuestionario Medimos Acceso y Calidad del Aborto (MACA). Argentina, 2023-2024.

Preguntas	Respondió Sí* (%)
¿Se sintió cómoda/e para expresar sus necesidades, preguntas y/o temores a quienes la/le atendieron durante el proceso de aborto?	88,7
Cuando compartió su información personal con quienes la/le atendieron, ¿sintió que mantendrían la confidencialidad?	93,4
¿Sintió que quienes la/le atendieron respetaron su decisión de abortar?	93,9
¿Sintió que quienes la/le atendieron la/le presionaron para abortar o para no abortar?	95,8
Quienes la/le atendieron, ¿le dieron información sobre las siguientes opciones de métodos para abortar: el aborto con medicamentos?	98,0
Quienes la/le atendieron, ¿le dieron información sobre las siguientes opciones de métodos para abortar: la aspiración manual endouterina conocida como AMEU?	74,3
Quienes la/le atendieron, ¿le dieron información sobre las siguientes opciones de métodos para abortar: el legrado o raspaje?	45,4
Quienes la/le atendieron, ¿le hablaron sobre la seguridad de los métodos para realizar el aborto?	97,2
Quienes la/le atendieron, ¿le dieron información sobre cómo usar los medicamentos? [En caso de realizar el aborto con medicamentos)	99,1
Quienes la/le atendieron, ¿le dieron información sobre qué iba a suceder durante la aspiración manual endouterina (conocida como AMEU) o el legrado/ raspaje?	93,3
Quienes la/le atendieron, ¿le dieron información sobre lo que podría sentir durante y después del aborto?	97,6
Quienes la/le atendieron, ¿le dieron información sobre los signos de alerta a los que tenía que estar atenta?	95,3
Quienes la/le atendieron, ¿le dieron información sobre cómo saber si se había completado el aborto?	90,5
Quienes la/le atendieron, ¿le dieron información sobre cómo aliviar el dolor durante el aborto?	95,8
Quienes la/le atendieron, ¿le dieron la información que necesitaba sobre el proceso de aborto?	93,9
Quienes la/le atendieron, ¿se aseguraron de que hubiera comprendido bien toda la información que le dieron?	95,3
Quienes la/le atendieron, ¿le preguntaron sobre el método de aborto (medicamentos/aspiración manual endouterina, conocida como AMEU/legrado) que quería?	78,1
¿Pudo tener el aborto con el método que quería?	94,8
¿Sabía qué hacer frente a un signo de alerta?	83,4
¿Le dieron o le recomendaron alguna medicación para aliviar el dolor durante su aborto?	96,7
¿La medicación que le dieron o le recomendaron le alivió el dolor?	62,5
Quienes la/le atendieron, ¿le transmitieron seguridad en las consultas sobre el aborto?	95,7
¿Quiénes la/le atendieron durante el proceso de aborto la trataron con respeto?	95,2
¿Se sintió acompañada/e por quienes la/le atendieron durante el proceso de aborto?	91,9
Quienes la/le atendieron durante el proceso de aborto ¿le dieron información sobre métodos anticonceptivos?	98,6
¿Recibió el método que quería? [Solo en caso de querer un método anticonceptivo, el afirmativo significa que lo recibió en el mismo momento]	81,8

Fuente: Elaboración propia. *Respondió “Sí, en todo momento”, o de otra forma positiva. Las categorías de respuesta variaron según la pregunta. Para más información se puede consultar el instrumento publicado^19^.

Además, calculamos las correlaciones de las respuestas entre las preguntas vinculadas a calidad para asegurar que siguieran patrones razonables y que no hubiera confusión. Todas las preguntas que se suponía debían tener correlaciones positivas o neutrales las tuvieron, así como aquellas que debían tener correlaciones negativas o neutrales. No hubo preguntas que correlacionaran más de 0,75, mostrando que las preguntas relevaron información diferente pero las respuestas estaban relacionadas (Tabla 4). Entre las tres preguntas con correlaciones más positivas, todas correlacionaron con una otra pregunta: “Quienes la/le atendieron, ¿se aseguraron de que hubiera comprendido bien toda la información que le dieron?” Esta pregunta correlacionó muy positivamente con la pregunta, “Quienes la/le atendieron, ¿le dieron la información que necesitaba sobre el proceso de aborto?”, “Quienes la/le atendieron, ¿le dieron información sobre lo que podría sentir durante y después del aborto?”, y “Quienes la/le atendieron, ¿se aseguraron de que hubiera comprendido bien toda la información que le dieron?” (Tabla 4).

**Tabla 4:** Correlaciones entre preguntas seleccionadas. Cuestionario Medimos Acceso y Calidad del Aborto (MACA). Argentina, 2023-2024.

Tipo de correlación	Pregunta 1	Pregunta 2	Correlación
Correlaciones más positivas entre preguntas de calidad	Quienes la/le atendieron, ¿le transmitieron seguridad en las consultas sobre el aborto?	Quienes la/le atendieron, ¿se aseguraron de que hubiera comprendido bien toda la información que le dieron?	0,69
	Quienes la/le atendieron, ¿le dieron la información que necesitaba sobre el proceso de aborto?	Quienes la/le atendieron, ¿se aseguraron de que hubiera comprendido bien toda la información que le dieron?	0,73
	Quienes la/le atendieron, ¿le dieron información sobre lo que podría sentir durante y después del aborto?	Quienes la/le atendieron, ¿se aseguraron de que hubiera comprendido bien toda la información que le dieron?	0,66
Correlaciones más negativas entre preguntas de calidad	Quienes la/le atendieron durante el proceso de aborto, ¿la trataron con respeto?	¿Sintió que quienes la/le atendieron la/le presionaron para abortar o para no abortar?	-0,26
	Quienes la/le atendieron, ¿le dieron la información que necesitaba sobre el proceso de aborto?	¿Sintió que quienes la/le atendieron la/le presionaron para abortar o para no abortar?	-0,26
	¿Se sintió acompañada/e por quienes la/le atendieron durante el proceso de aborto?	¿Sintió que quienes la/le atendieron la/le presionaron para abortar o para no abortar?	-0,27

Fuente: Elaboración propia.

Después de analizar todas las respuestas, las únicas preguntas que decidimos modificar fueron las de elegibilidad, para asegurar la claridad, según sugirieron las personas profesionales de salud que invitaron a las personas encuestadas. 

Como instrumento complementario y con la intención de facilitar el proceso de transferencia de esta encuesta, se elaboró un manual de uso^20^ y se diseñó el instrumento en Google Forms para que estuviera disponible para cualquier persona que quisiera aplicar la encuesta en un servicio de salud (ver el material suplementario). 

## DISCUSIÓN Y CONCLUSIONES

La Ley 27610 fue producto de una larga historia de activismos, alianzas e incidencia legislativa^2^^,^^21^. Ello hizo de la IVE una ley de alto perfil, sostenida por una masiva movilización social y el apoyo del espectro partidario y, a su vez, sometida a posicionamientos ideológicos muy fuertes que hacía necesario el monitoreo de su implementación como política pública. 

La OMS define el monitoreo como el proceso de observar repetidamente una situación para detectar cambios a lo largo del tiempo^22^. Medir la accesibilidad y calidad de la atención en Argentina es una forma de monitoreo centrada en el nivel microsocial de los servicios de salud. Como es sabido, la atención de calidad debe estar centrada en las personas usuarias, responder a sus expectativas y satisfacer su derecho a la salud^23^.

Comprender las experiencias de las personas usuarias con los servicios de aborto permite evaluar el desempeño de los equipos de salud, identificar áreas de mejora e informar a las personas tomadoras de decisión sobre las dificultades para navegar el sistema. Además, estos datos pueden ayudar a identificar desigualdades en los distintos territorios y entre poblaciones con diversas características y necesidades. Con estos objetivos, hemos desarrollado y validado un instrumento para medir la accesibilidad y la calidad de los servicios de aborto en Argentina.

En el desarrollo del instrumento buscamos, en primer lugar, alcanzar la validez de contenido. Una vez que identificamos los estándares internacionales para medir calidad de la atención, estos necesariamente debían traducirse, adaptarse y validarse en nuestro contexto. Consideramos la validez de personas expertas, como voces calificadas sobre las características del sistema de salud y la población usuaria en diversas regiones del país. También consideramos la validez de la comprensión del instrumento, que requiere que las personas participantes interpreten una determinada frase y respondan a ella^18^.

Entre las fortalezas del estudio resaltamos, en primer lugar, esta metodología de consulta con actores claves del campo, utilizada para garantizar la validez teórica, técnica y política del cuestionario MACA como un instrumento relevante para el contexto argentino y válido para ser aplicado por otros equipos. En segundo lugar, la prueba piloto testeó el instrumento y sus indicadores en diversos territorios y servicios y proporcionó evidencia sobre su factibilidad y confiabilidad. Como resultado, el cuestionario MACA incluye indicadores que representan diversos tipos de accesibilidad de los servicios (simbólica, organizacional, geográfica y económica) y diversos dominios de la calidad de la atención (interacción entre persona usuaria y quienes la atendieron, provisión de información, toma de decisiones y competencias técnicas).

El cuestionario MACA fue diseñado para ser aplicado en los tres subsectores del sistema de salud de la Argentina. En esto se diferencia de la herramienta ACQTool, que fue diseñada para medir calidad también en servicios comunitarios, farmacias, y líneas telefónicas^15^. Estas diferencias contextuales y el propósito específico de monitorear la reciente implementación de la política de acceso al aborto en Argentina, resultó en algunas decisiones sobre indicadores diferentes a los de ACQTool. Así fue que incluimos indicadores de accesibilidad simbólica (grado de conocimiento de la población usuaria sobre la legalidad del aborto y dónde solicitarlo); indicadores sobre las vías de acceso (cantidad de visitas, cantidad de servicios) y sobre la accesibilidad geográfica (tiempo de viaje), así como indicadores subjetivos para medir las expectativas sobre la atención (aceptabilidad del tiempo transcurrido y de la cantidad de visitas a servicios de salud hasta recibir la atención). Desde una perspectiva interseccional, decidimos desde el principio incluir preguntas sobre la identidad de género, la discapacidad, la identidad indígena o afrodescendiente, la migración, el lugar de residencia en la ciudad capital o en el interior de cada provincia, el nivel educativo, la edad y la ocupación de las personas usuarias con el fin de describir cómo varían la accesibilidad y calidad de la atención según múltiples ejes de desigualdad social que se intersectan y coproducen situaciones de mayor o menor vulnerabilidad. 

Al mismo tiempo, tuvimos en cuenta las prácticas locales y los estándares establecidos por la ley. En nuestro contexto, resulta relevante registrar la cantidad de días desde la solicitud del aborto hasta su resolución para relevar la adhesión a la norma, que establece un plazo máximo de 10 días. En el mismo sentido, reemplazamos un indicador subjetivo, como es el grado de asequibilidad de los servicios para las usuarias, por la indicación de cualquier gasto de bolsillo para acceder a la práctica de IVE-ILE a fines de relevar el cumplimiento de la cobertura integral y gratuita de los servicios, tal como indica la ley^21^. Reconociendo que la erradicación del legrado es todavía una deuda pendiente en el país, a pesar de ser una tecnología explícitamente no recomendada por la OMS ni por el protocolo nacional, decidimos preguntar por cada uno de los métodos de aborto por separado e identificar dónde se practican legrados para eventualmente impulsar capacitaciones para incorporar las técnicas adecuadas^3^^,^^28^^,^^29^.

La publicación en acceso abierto del *Manual para la aplicación de la Encuesta MACA*^20^, como así también la publicación del cuestionario como material suplementario de este artículo, busca transferir este instrumento para que sea utilizado en diferentes ámbitos. Entre sus limitaciones podemos señalar que la prueba piloto se realizó en seis provincias, aunque la Encuesta MACA se diseñó como una herramienta de monitoreo de alcance nacional. El acceso a la población objetivo, es decir a las personas usuarias de los servicios de aborto, se realizó por medio de profesionales de la salud, quienes las invitaron a participar. Sabemos que esto podría representar un sesgo de cordialidad de las usuarias para con quienes las habían atendido, aun cuando se trataba de un cuestionario anónimo y autoadministrado por las personas usuarias desde sus propios dispositivos, en el lugar y el momento que eligieran. 

Aunque la calidad de la atención se definió de manera amplia y teniendo en cuenta múltiples dimensiones, no todas ellas están incluidas en el instrumento. El cuestionario MACA no releva los sistemas de referencia y derivación de los servicios de salud ni la gestión de los insumos. Tampoco evalúa los resultados clínicos de la atención (efectividad, incidencia de complicaciones u otros eventos de salud), ni la perspectiva de las y los profesionales de la salud y su potencial sobrecarga laboral y emocional, entre otros aspectos. Además, el cuestionario MACA releva únicamente la calidad de la atención en referencia a quienes atendieron a las personas usuarias en los lugares donde efectivamente accedieron a un aborto y las invitaron a participar. De esta forma se excluyó al personal de recepción o administrativo y de otros servicios (ecografía, derivaciones, así como rechazos y atenciones inconducentes). Por lo tanto, salvo que se trate de una intervención pública centralizada para mejorar la calidad del aborto, es probable que el cuestionario sea aplicado por personas e instituciones comprometidas con la atención de calidad y que los resultados observados sean mejores en esos sitios que en el universo de los servicios de salud sexual y reproductiva del país.

Sin embargo, a partir de los temas más relevantes identificados por los paneles de personas expertas, el cuestionario MACA tiene importantes puntos en común con estándares internacionales y refleja las cuatro dimensiones de calidad que han sido validadas en múltiples contextos globales^6^. También, la importancia dada a los indicadores sobre la contención y el acompañamiento concuerda con otras investigaciones en Argentina, que muestran el valor que las personas usuarias otorgan al apoyo, la confianza y el acompañamiento a largo plazo durante el proceso de aborto^10^^,^^28^^,^^30^. Este conjunto de dimensiones reconocidas como importantes a nivel internacional, así como el enfoque y la adaptación al contexto argentino, garantizan que el instrumento sirva para medir apropiadamente dimensiones claves de accesibilidad y calidad. Por último, el cuestionario MACA se propone como una herramienta para el monitoreo de la garantía de los derechos sexuales y (no) reproductivos reconocidos en la ley argentina. Esperamos pueda ser de utilidad para que activistas, equipos de salud y decisores de políticas públicas lo apliquen con el fin de lograr un mejor desempeño de la política de aborto en Argentina y una mayor satisfacción de las personas usuarias del sistema de salud.

## MATERIAL SUPLEMENTARIO

Cuestionario diseñado en Google Forms para la Encuesta MACA (Medimos Acceso y Calidad del Aborto), disponible en línea (ver el Material suplementario.)
