# Supplementary material for: Diseño y validación de un cuestionario para medir acceso y calidad de los servicios de aborto en Argentina
Source: Salud Colect. 2025 Apr 22;21:e5348. [Article in Spanish] doi: 10.18294/sc.2025.5348 (PMC12309473; doi:10.18294/sc.2025.5348)
Supplement: Translation

# Translation: Design and validation of a questionnaire to measure access to and quality of abortion services in Argentina

**Authors:** Brianna Keefe-Oates, Mercedes Krause, Agustina Ramón Michel, Silvina Ramos, Mariana Romero

**Affiliations:** 1 PhD in Population Health Sciences. Postdoctoral researcher, Roux Institute, Northeastern University, Maine, USA. bkeefeoates@gmail.com; 2 PhD in Social Sciences. Researcher, Centro de Estudios de Estado y Sociedad, Instituto de Investigaciones Gino Germani, Universidad de Buenos Aires, Ciudad Autónoma de Buenos Aires; Argentina. merkrause@gmail.com; 3 Master in Law. Associate Researcher, Centro de Estudios de Estado y Sociedad, Instituto de Investigaciones Gino Germani, Universidad de Buenos Aires, Ciudad Autónoma de Buenos Aires, Argentina. rmichelagus@gmail.com; 4 Sociologist. Researcher, Centro de Estudios de Estado y Sociedad, Instituto de Investigaciones Gino Germani, Universidad de Buenos Aires, Ciudad Autónoma de Buenos Aires, Argentina. silvinaramosarcoiris@gmail.com; 5 Master in Reproductive Health. Independent Researcher, Consejo Nacional de Investigaciones Científicas y Técnicas. Researcher, Centro de Estudios de Estado y Sociedad, Instituto de Investigaciones Gino Germani, Universidad de Buenos Aires, Ciudad Autónoma de Buenos Aires, Argentina. mromero@cedes.org

**Keywords:** Abortion, Sexual Health, Reproductive Health, Surveys and Questionnaires, Sanitary Monitoring, Argentina

## Abstract

In Argentina, Law 27610, which legalized voluntary termination of pregnancy, was the result of years of activism and political alliances. To monitor its implementation, between 2022 and 2024 an instrument was developed to assess the accessibility and quality of abortion services, considering users’ experiences. We conducted a three-phase study that included expert panels, cognitive interviews, and a pilot test of the instrument with individuals who had undergone abortions. These efforts led to the creation of the questionnaire Measuring Accessibility and Quality in Abortion Services (MACA), which can be applied within the Argentine health system. This questionnaire can be used for monitoring purposes, to identify areas for improvement in health services, and to detect inequalities in access based on geographic and social characteristics.

## INTRODUCTION

On December 30, 2020, the Argentine National Congress passed Law 27610 on Access to Voluntary Termination of Pregnancy, thus concluding a long process of social mobilization and political anticipation. This legal milestone was accompanied by the societal phenomenon of the “green wave,”^1^ which extended as a source of inspiration across Latin America and beyond. From that point until December 2023, the implementation of a national public policy ensuring access to abortion was grounded in two pillars: the distribution of abortion supplies to all provinces and the public production of misoprostol to ensure its availability and financial accessibility.^2^

In 2023, through Regulation 1470/2023, the National Administration of Drugs, Food and Medical Technology (ANMAT) authorized the registration of mifepristone, a medication that expands therapeutic options and enhances the safety and efficacy of medication abortion, in line with international recommendations from the World Health Organization (WHO).^3^ In addition, the National Directorate for Sexual and Reproductive Health, the governing body under the Ministry of Health, developed more than 40 documents to guide policy implementation and clinical practice. These contributed to strengthening coordination with provincial governments and fostering a secure and predictable working environment for healthcare teams.

Training sessions on manual vacuum aspiration (MVA) were also conducted, involving nearly 500 professionals between 2021 and 2023, thereby broadening the range of available abortion methods. As a result, the number of public health services guaranteeing access to voluntary and legal termination of pregnancy (VTP-LTP) doubled, increasing from 907 in 2020 to 1,982 in 2023. Meanwhile, the number of medication abortion treatments distributed increased ninefold, from 18,590 in 2020 to 166,164 in 2023.^4^

This process of institutionalizing and implementing the Law on Access to Voluntary Termination of Pregnancy in Argentina introduced, for the first time on a large scale and throughout the health system, reproductive technologies such as misoprostol and mifepristone, along with care protocols aligned with international standards. These represent historic changes in the accessibility and quality of abortion care services, and require ongoing monitoring to ensure that the rights recognized by law are effectively guaranteed in the lives of women and other pregnant individuals, and that the changes in care delivery improve the experiences of people seeking abortion care.

Quality of abortion care is a critical dimension of abortion policy that has been recently emphasized by the World Health Organization (WHO), yet there are no standardized indicators available in Argentina^3^ to assess it. Understanding this dimension and examining its characteristics within health services is a key gap in abortion policy monitoring that requires specific measurement tools.

The academic literature shows a lack of consensus regarding the appropriate standards for measuring the quality of abortion services. In a review of indicators used between 2008 and 2018 to evaluate progress in abortion care, Filippi *et al*. identified nearly 800 indicators, of which only 22% were used consistently across sources, and most related to the prevalence and incidence of abortion.^5^

Quality of care is rarely measured from the perspective of people seeking abortion in Latin America.^6^ Some exceptions include the evaluation by Billings and Benson on Latin America,^7^ pre-Law 27.610 studies on postabortion care quality in public hospitals in Tucumán and the Autonomous City of Buenos Aires,^8^^,^^9^ and a qualitative study on the preferences and priorities of people undergoing abortions regarding their interactions with accompaniers and healthcare professionals in Buenos Aires and Neuquén.^10^ More commonly, quality-of-care questionnaires focus on abortion providers or healthcare services, gathering information on structural dimensions such as infrastructure, wait times, or technical competence in effective pain management.^5^^,^^10^^,^^11^ When patients are taken into account, satisfaction is generally measured using scales.^12^ This type of instrument tends to yield high satisfaction rates, even in contexts where quality of care is low. As a result, the validity of satisfaction levels as an indicator of abortion care quality has been questioned.^13^^,^^14^

In this context, in 2018, the international organizations Metrics for Management (M4M), Ibis Reproductive Health, and Ipas collaborated to launch the Abortion Service Quality Initiative (ASQ) and developed the first global standard: the Abortion Care Quality Tool (ACQTool), which measures the quality of abortion services in low and middle-income countries.^12^^,^^15^

Aligned with these efforts, “Proyecto Mirar”-an initiative of the Centro de Estudios de Estado y Sociedad (CEDES) and Ibis Reproductive Health that monitors the implementation of the Law on Voluntary Termination of Pregnancy in Argentina - developed, over the past three years, the questionnaire *Measuring Accessibility and Quality in Abortion Services* (MACA). The goal was to create an instrument to assess the accessibility and quality of abortion services that could be widely used throughout the health system. The study aimed to design and validate an anonymous, self-administered questionnaire to evaluate accessibility and quality of care following legalization. In line with the ACQTool and in consultation with a range of key actors and stakeholders in Argentina, the MACA study sought to develop a context-specific and useful tool. In other words, the MACA questionnaire is a standardized instrument for measuring access to and quality of abortion services from the perspective of people seeking abortion within the healthcare system, tailored to the Argentine context and the current Law on Voluntary Termination of Pregnancy.

Like the ACQTool, the MACA questionnaire measures quality of care from the perspective of patients, focusing on their subjective experiences, the care processes, and their perceptions of the services received. Considering these aspects is essential for understanding the variability of concrete experiences when accessing abortion services. People’s perceptions of care can influence beliefs about abortion and affect public trust in the health system.

The objective of this article is to describe the development process of the MACA questionnaire, a standardized instrument designed to measure access to and quality of abortion services from the perspective of patients, specifically tailored to the Argentine context and current abortion legislation. We aim to make explicit the decisions and procedures undertaken to ensure the instrument’s validity and reliability, defined respectively as the degree to which the tool measures what it is intended to measure, and whether it does so consistently. In particular, we present evidence regarding three types of validity: 1) content validity, that is, the extent to which the instrument reflects access and quality of services according to the domains associated with these concepts in the academic literature; 2) expert validity, or the degree to which the instrument captures the construct as understood by subject matter experts; and 3) validity related to by those who may respond to the instrument.

## METHODOLOGY

This study used a mixed-methods approach, carried out in three stages to develop a questionnaire. These stages included: 1) expert panels to identify priority indicators related to access and quality (including both questionnaires and discussion groups); 2) interviews with experts to gather feedback on the instrument; and 3) a pilot test of the quantitative instrument with individuals who had an abortion (Figure 1).

**Figure 1:** Steps in the development of the Measuring Accessibility and Quality in Abortion Services (MACA) questionnaire. Argentina, 2023-2024.

Source: Own elaboration.

### Stage 1: Expert panels

The aim of this stage was to identify a list of relevant and necessary indicators to measure access to and quality of abortion services in the Argentine context. We adapted the nominal group technique and the Delphi method to construct the list of indicators that the instrument would measure, with the goal of identifying barriers and gaps in access to and quality of abortion services from the perspective of the individuals using them.^16^^,^^17^ Through the expert panels, we gathered opinions and knowledge from a diverse group of participants to reach a consensus on which of the proposed indicators were most relevant for measuring each dimension of accessibility and quality. In doing so, we sought to establish *expert validity*.^18^

We started with 42 indicators, aiming to reduce this list to those that were relevant and highest priority for the Argentine context. We based our initial list on the abortion service quality indicators standardized by the ACQTool and the literature on accessibility and quality of healthcare services in general, and abortion services in particular.^24^ As a bilingual team, with Argentine and U.S. researchers with expertise in sexual and (non) reproductive health, we translated the indicators from English to Spanish and partially adapted the international proposals. These indicators were then reviewed by the expert panels to ensure correct grammar and appropriate alignment with the local context.

We also reviewed the information system of the *Proyecto Mirar*, to help determine important indicators. This system, prior to the development of the MACA questionnaire, had identified gaps in the available secondary sources for measuring actions and changes in the implementation of the public abortion access policy. This strategic selection process for indicators within the monitoring information system of *Proyecto Mirar* was based on two widely recognized evaluation frameworks for identifying and conceptually defining specific, measurable, and feasible indicators: the Monitoring Results for Equity (MoRES) and the Availability, Accessibility, Acceptability, Quality (AAAQ) frameworks.^25^^,^^26^^,^^27^

The initial list of indicators for the MACA questionnaire instrument, which served as the starting point for the consultation process with experts, included indicators corresponding to five dimensions: one for access and four for quality. The quality dimensions, adapted from those identified in the ACQTool, included: support/accompaniment/psychological care, provision of information, decision-making, and technical competence.

The expert panels were organized in two phases: a questionnaire regarding the perceived relevance of each the proposed indicators and three discussion groups to facilitate discussion about the indicators for which there was no consensus based on the questionnaire results.

We invited a purposive sample to participate in the panel. The participants included three professional profiles involved in the management and provision of abortion services: a) abortion service providers; b) leaders of women’s organizations; and c) decision-makers involved in the implementation of Law 27.610. All the experts had at least five years of experience working in the field of abortion services.

In the service provider group, we sought to represent both the public health sector (covering the provision of services at primary, secondary, and tertiary care levels), the private sector, and the community sector (those who accompany individuals who self-manage their abortion outside the healthcare system). In the women’s organizations group, we aimed to include organizations focused on territorial advocacy and supporting individuals seeking abortions. In the decision-maker group, we invited individuals involved in the management of policies, programs, and services at national, provincial, and municipal levels. Additionally, we made efforts to ensure geographical diversity in each group, forming three groups with 10 to 11 participants each.

Initially, we administered a questionnaire through the Qualtrics platform to the experts who consented to participate. This questionnaire included a list of indicators previously selected by the research team. Participants were asked to evaluate the relevance of each indicator in measuring accessibility or quality by assigning a value from 1 (representing “Not relevant”) to 5 (representing “Very relevant”).

Once the questionnaires were completed, we developed a system to identify indicators with majority agreement for inclusion or elimination (indicators with scores reflecting a high degree of agreement among experts) and those without consensus.

We selected the indicators for the discussion groups following these steps and criteria:

We identified the indicators whose scores showed high agreement and would be directly included in the instrument without the need for discussion groups because: 1) all responses rated them between 3 and 5 (from neutral to “very relevant”), or 2) at least 92% of the experts rated them as 4 (relevant) or 5 (very relevant). We also included indicators from the ACQTool that we considered did not duplicate any other indicator.

We decided to discard the indicators with less support among the experts: less than 80% of the experts rated the indicator as “relevant” or “very relevant,” or between 80% and 91% of the experts chose “relevant” or “very relevant,” but the average of all responses was in the lowest third of the responses, because a group of respondents rated them as not relevant.

The remaining indicators (those for which there was no clear consensus regarding their inclusion or exclusion from the instrument) were discussed during the focus groups.

Once the quantitative data was gathered, we proceeded to share a results report via email with the experts, identifying the areas of agreement and disagreement in their responses. We then facilitated for three virtual focus groups, one with each subgroup of experts, all of whom had previously answered the questionnaire. A total of 27 people participated in the focus groups; between 9 and 11 people in each discussion.

We began the groups with a brief presentation of the results to ensure that everyone had the necessary information, and then guided them in an open discussion focused on each of the indicators where there was no consensus according to the questionnaire results. Organized thematically, these indicators were displayed on the screen alongside the indicators that had been directly included, and participants were asked to reflect on their content: Is this already addressed by another indicator? Why would it be necessary to include this indicator in the final instrument? At the end of the discussion for each indicator, we sought to reach a consensus on whether or not to include the indicator based on the arguments presented. In some cases, the group suggested modifications or a new indicator, which was brought forward as a suggestion to the next group for iterative discussion.

These groups were held via Zoom and recorded, with prior consent. All participants received a voucher for a bookstore as a recognition of their participation. Two researchers reviewed the recordings to document the opinions on each indicator that emerged in each group. The research team as a whole reviewed these suggestions. In cases of disagreements between the groups, we incorporated the suggestion when two of the three groups agreed, or based on our own expertise when the suggestions and modifications could not be endorsed by the previous groups, or when we recognized that the indicator was relevant and could not be collected from another data source.

### Stage 2: Interviews with key informants

After determining the indicators for the instrument, the research team developed a preliminary instrument containing questions aligned with the selected indicators. For each indicator from the Abortion Service Quality (ASQ) initiative, we used the corresponding question, adapting the language to fit the local context. For indicators without ASQ-defined questions, we adapted items from previous studies and/or formulated new ones.

The instrument included 54 questions. Seventeen questions focused demographic questions and the abortion experience in order to capture differences in accessibility and quality based on sociodemographic characteristics and individual experiences (e.g., by healthcare subsector or gestational age). In addition, 11 questions addressed access indicators, and 26 corresponded to the quality indicators established through expert panels.

Once the instrument was structured, we sought to validate the content and wording of the questions. Using the technique of cognitive interviewing for questionnaire content validation, we conducted interviews with qualified informants, as logistical and ethical considerations prevented us from conducting cognitive interviews with individuals who had recently had an abortion.^19^ Interviewees included members of women’s organizations that support people seeking abortion services, abortion providers, and academics with at least two years of experience in the field. We used purposive sampling based on our knowledge of the field, selecting individuals who represented a diversity of experiences with abortion services across various provinces. None of the interviewees had participated in the focus groups.

The nine interviewees were contacted via email and, upon agreeing to participate, received a draft version of the instrument to review in advance. Interviews were conducted via Zoom. After obtaining informed consent, we asked participants to take on the perspective of a patient, read the questionnaire items, and consider how they would respond after having had an abortion. In each section, we asked them to identify any confusing questions or response categories and to suggest alternatives. We also explored a subset of questions and response categories in greater depth to confirm their interpretations. At the end of the interview, we inquired about vocabulary preferences, including the use of “IVE/ILE” instead of “abortion,” how to refer to the individuals completing the questionnaire (e.g., patients, people seeking abortions), and preferred verbs associated with abortion, such as “to have,” “to obtain,” or “to undergo” an abortion. Each participant received financial compensation in recognition of the time dedicated to the interview.

The interviews were recorded and notes were taken. Based on this material, a table was compiled with the changes suggested by each participant. The full research team then reviewed the suggestions and modified the instrument accordingly.

### Stage 3: Pilot testing of the instrument

A pilot test of the instrument was conducted with individuals who had accessed abortion services in healthcare facilities in Argentina, with the following objectives:

- To evaluate the feasibility of self-administering the instrument and the necessary procedures to enable this.
- To validate the questionnaire items and identify any necessary revisions.
- To standardize logistical procedures for administering the instrument.
- To analyze preliminary data to assess the quality and accessibility of services where the pilot was conducted.

The pilot was carried out in 11 healthcare facilities between June 2023 and April 2024 (Table 1), selected intentionally to represent a range of geographic and health system contexts, as well as diverse experiences related to abortion methods and types of facilities. A fieldwork manual was developed, and healthcare professionals who provide abortion services were trained in the content of the questionnaire and the procedures for identifying eligible patients and collecting data.

**Table 1:** Health facilities participating in the pilot test of the “Measuring Access and Quality of Abortion” (MACA) questionnaire. Argentina, 2023-2024.

Characteristics	Number of services (n=11)
**Province of the facility**	
Jujuy	4
Santa Fe	3
Buenos Aires	1
Entre Ríos	1
Salta	1
Neuquén	1
**Type of facility**	
Hospital	5
Primary healthcare center	4
Clinic	2
**Health subsector**	
Public	9
Private	1
Social security	1
**Abortion method offered**	
Medication and MVA	5
Medication only	4
MVA only	2

Source: Own elaboration. MVA = Manual vacuum aspiration.

To be eligible, participants had to be at least 16 years old, have undergone an abortion within the past three months at one of the facilities selected for the pilot, be able to speak and read Spanish, and must not have had an abortion on their own or solely with the accompaniment of a feminist or community group without having first received care from the selected facility. Once the abortion was completed and the person had been medically discharged, healthcare professionals at each institution - who had been trained as part of the study - invited patients to participate in the survey. As part of the invitation process, each professional provided information about the study’s objectives, procedures, and assurances of anonymity and confidentiality.

Interested participants were offered an informational leaflet about the study that included a QR code and a link to the online questionnaire, which was designed using the encrypted platform Qualtrics. Scanning the code led to a set of questions confirming informed consent and participant eligibility, after which they gained access to the survey.

The survey included sociodemographic questions as well as items corresponding to the indicators identified through expert panels and refined via cognitive interviews. Completing the survey took approximately 15 to 20 minutes.

We conducted a descriptive analysis of the sample characteristics and of the questions related to access and quality, with the aim of exploring the instrument’s validity and identifying patterns of missing responses. Since the questions on quality focused primarily on perceptions and were grouped into four dimensions, we analyzed the extent to which these items reflected the thematic areas the instrument was designed to measure. We conducted statistical analyses to assess the frequency of responses and determine whether the items could detect meaningful differences in perceived quality. We then examined correlations among the quality-related items to assess whether: 1) thematically opposing items were negatively correlated as expected, and 2) whether any highly correlated items appeared to reflect the same theme and, therefore, did not both need to be included in the instrument.

We also gathered feedback from the healthcare professionals involved in data collection to better understand the feasibility of administering the questionnaire in real-world settings and to adjust procedures based on their input. Changes were made accordingly to produce the final version of the instrument.

The research project was approved by the ethics committees and/or authorities of each of the participating institutions. It was also reviewed and approved by the Research Ethics Committee “Iniciativa y Reflexión Bioética,” affiliated with Respire Centro Médico, and the following provincial committees: the Provincial Health Research Ethics Committee of the Government of Jujuy (Ref. file: 773-1251/2023); the Research Ethics Committee of the Public Health Secretariat of the Municipality of Rosario; and the Provincial Commission on Biomedical Research of the Ministry of Public Health of Salta (Ref. file: 244-160192/2023-0 and Code 1). Information regarding each committee and institutional approval is not included in order to protect the anonymity of the services where data collection took place. All data were anonymized and stored in protected files.

## RESULTS

### Stage 1: Expert panels

#### Phase one: Survey of experts

A total of 35 experts were selected according to the specified criteria. Based on the analysis described above, we identified 17 indicators that were directly included as final indicators for measuring access and quality, 13 indicators to be discussed in focus groups, and 12 that were excluded based on the survey results and deemed not warranting further discussion. In general, the indicators selected through the expert survey helped focus the instrument on issues directly related to abortion, while leaving out others with a more tangential relationship, such as those concerning contraceptive services. Both objective and subjective measures were considered relevant by the experts. For instance, respondents were asked to report the number of days between requesting and receiving the abortion, as well as their perception of the acceptability of that wait time (longer than expected, as expected, or shorter than expected).

When analyzing the survey results on access indicators, there was consensus on excluding two indicators related to out-of-pocket expenses: “The cost of transportation to the provider is affordable for the patient,” and “The patient paid out-of-pocket for domestic and/or caregiving services during the abortion process.” However, two other indicators directly associated with the cost of the abortion procedure were considered relevant - though without full consensus - and were therefore addressed in the discussion groups: “The patient paid out-of-pocket for the ultrasound required for the abortion,” and “The patient paid out-of-pocket for the medication used in the abortion.”

From the analysis of the survey responses regarding quality indicators, two indicators from the “Support/Emotional Care/Accompaniment” dimension were discarded. One of these, “Patients felt comfortable sharing personal information with the staff,” was excluded due to its similarity to “Patients trusted that the provider would maintain the confidentiality of their personal information,” the latter receiving stronger support. The indicator “Providers refer and give patients complete information about other relevant services, as needed (e.g., sexual and reproductive health services, legal services, gender-based violence, etc.)” was excluded because it focused on broader issues beyond abortion. Additionally, under the “Provision of Information” dimension, the indicator “Patients knew what to expect at each step of their visit/care provision” was discarded due to its overlap with other indicators in the same dimension.

#### Phase two: Discussion groups

In the discussion groups, participants focused on deciding whether or not to include certain indicators and, in some cases, suggested the addition of new ones. They also provided feedback on the wording of items. In general, there was agreement on discarding indicators related to expectations and retaining those addressing patients’ perceptions of the actions taken by the healthcare team.

The groups also proposed indicators that had not previously been considered or had been removed based on the survey results. For example, although the questionnaire results showed less interest in measuring geographic accessibility through a transportation-related indicator, the topic was mentioned several times in the discussion groups. As a result, a new indicator on geographic barriers - measured as travel time to the abortion services visited - was added as a measure of accessibility. A more comprehensive indicator on out-of-pocket expenses was also suggested, as this was identified by participants as an important dimension of accessibility. Finally, another topic raised was the importance of receiving information about all available methods for abortion - an indicator that had not been previously included.

All discussion groups made distinctions between support, accompaniment, and relief experienced by patients seeking an abortion. Within this exchange, recommendations emerged to assess these aspects separately, as they could reflect different dimensions of quality. While some participants considered that support and accompaniment were related to the emotional care received during the process, others interpreted support as something received at a specific moment during the consultation, whereas accompaniment could be understood as continuous presence throughout the entire abortion process. Relief, according to participants, was associated with achieving the abortion itself; patients may feel relieved to have “resolved” the abortion, even without appropriate accompaniment. Following these discussions and after reviewing other indicators to avoid redundancy, a new indicator was selected: “Patients felt accompanied throughout their abortion process.” This was considered more appropriate to capture both support and accompaniment over time, while other indicators addressed specific types of support (e.g., respect) at the moment of receiving care in the health facility.

Finally, during the discussion groups, suggestions were made to improve the precision of the sociodemographic data to be collected and the terminology used in the questionnaire. For example, in two discussion groups, participants emphasized the importance of collecting information about the health subsector where care was received, to allow for quality assessments by subsector. In another group, specific recommendations were made regarding wording: using the term “patients” to ensure inclusive language, and specifying “throughout the entire abortion process” when asking about experiences, to avoid referring to a single moment.

#### Phase three: Final selection of indicators

In many cases, there was agreement among the three groups regarding whether to include or exclude a given indicator. Disagreements between groups were mainly about the wording of an indicator, rather than the underlying topic. In most cases, the research team incorporated the suggested changes in wording. In cases where there was no agreement among all groups, we selected the version endorsed by two out of the three groups. This process resulted in a total of 25 indicators (Table 2), grouped into five dimensions under the broader categories of access and quality, which were then used to develop the final instrument.

**Table 2:** Final indicators for measuring access and quality of abortion services in Argentina. “Measuring Access and Quality of Abortion” (MACA) questionnaire. Argentina, 2023-2024.

Category	Dimension	Final Indicator	Source
Access	Access	The patient knew where to go to request an abortion	Developed by the research team
		Number of days between the first request for an abortion and when The patient obtained the abortion or received abortion medication	ACQTool
		The waiting time from the first request for abortion services to obtaining the abortion or receiving medication was acceptable to Patients	ACQTool
		The patient knew they could request an abortion from a public, private, or social security health service	Developed by the research team
		The patient had any out-of-pocket expenses related to the abortion (e.g., ultrasound, medication)	Developed during focus group discussions
		Number of times The patient had to visit a health facility before obtaining the abortion	Developed during focus group discussions
		Total travel time from the moment the abortion was requested to when it was obtained (includes waiting times for transport)	Developed during focus group discussions
Quality	Support	Patients received information about the abortion process in line with their expectations	mirar project information system
		The patient felt respected at all times	ACQTool
		Patients trusted that the provider(s) would maintain the confidentiality of their personal information	ACQTool
		Patients felt supported throughout their abortion process	Developed during focus group discussions
	Information	The provider verified that Patients understood the information they received	ACQTool
		Patients knew what to do if they experienced warning signs or complications	ACQTool
		Patients knew how to determine whether their abortion was complete	ACQTool
		Among patients who wanted information on contraception: the provider offered complete information on contraception	ACQTool
		The patient received all the information about what would happen during their abortion	ACQTool
		The patient felt comfortable expressing their needs, questions, and fears to the provider(s)	ACQTool
		The patient received and understood information about the safety of all available abortion methods	ACQTool
		The patient received information about the range of available abortion methods	Developed during focus group discussions
	Decision-making	The patient felt that the provider(s) supported their decision to have an abortion	ACQTool
		The patient felt that the provider(s) did not pressure them either to have or not to have an abortion	Developed during focus group discussions
		Patients received the abortion method they wanted	ACQTool
		The patient received the contraceptive method they wanted after the abortion	ACQTool, mirar project information system
	Technical competence	The provider conveyed a sense of safety to The patient during the abortion procedure	ACQTool
		Patients felt that their pain was managed effectively	ACQTool

Source: Own elaboration.

### Stage 2: Instrument development and cognitive interviews

To ensure that the language and format of the instrument were clear and comprehensible, we conducted nine cognitive interviews between September and October 2022.

Participants provided suggestions regarding the wording and structure of the instrument, and in some cases proposed additional questions. We only modified items corresponding to indicators already selected by the expert panels, given the rigor of that prior process. One significant change was the decision to use the formal second person singular (“*usted*”) instead of the informal (“*vos*”), in order to increase the questionnaire’s acceptability across all provinces. The following specific suggestions were incorporated:

#### Clarifications

- Ensure clarity about which health service the questions refer to, as respondents may have visited multiple services (the instrument does not explore those other services). The following instruction was added at the beginning of the section “about your experience with those who cared for you during the abortion process”: “When we refer to the ‘abortion process,’ we mean from the moment you first sought care at the facility where you had the abortion until the procedure was completed. Please do not include experiences with other professionals or health services where you went or called but did not obtain the abortion. If you received care at more than one facility, please respond based on the one that invited you to participate in this survey.”
- Add response options for out-of-pocket expenses related to messaging services and blood tests; separate MVA (manual vacuum aspiration) and curettage instead of grouping them under “surgical procedure.”
- Clarify the timeframe of each question (e.g., “during the abortion process,” “during the consultations about the abortion”) and define what is meant by “the entire process.”

#### Question wording and format

- Avoid using grids in the survey. The grid concerning the amount of information received on various topics (e.g., how to use the medication, what to expect during the procedure) was converted into a block of individual questions.
- Disaggregate the response categories for abortion method to separately list MVA and curettage (instead of using the term “surgical method”).

#### Add the response option “I’m not sure.”

- Participants confirmed the appropriateness of using the term “to have the abortion” (e.g., “how many weeks pregnant were you when you had the abortion,” “safety of the methods used to have the abortion”).
- The word abortion was viewed more favorably than VTP/LTP (Voluntary or Legal Termination of Pregnancy), which was considered more formal and less accessible.
- Participants supported the use of “patients” instead of “patients” or the phrase “adolescents, women, and people with the capacity to become pregnant.”
- The use of the personal pronoun “usted” to address patients was confirmed in order to ensure the instrument’s validity throughout Argentina.

### Stage 3: Instrument pilot testing

#### Recruitment process

Between July and October 2023 and February and April 2024, individuals who had accessed abortion services in the previous three months at selected facilities were invited to participate in the survey.A total of 506 people were invited, of whom 254 responded and met the eligibility criteria. Ultimately, 225 individuals fully or partially completed the questionnaire. These results represent a response rate - calculated based on those who responded to the invitation and met the eligibility criteria - of 44.5%.

Most participants resided in Jujuy (36.8%), Santa Fe (18.2%), Buenos Aires Province (16.4%), and Entre Ríos (9.8%). Ages varied, but the majority were 26 years or older (58.6%). A total of 51.1% had completed tertiary education or higher, and 30.2% had completed secondary education. The majority had children (55.6%), and 58.2% relied solely on public health coverage. While 31.6% had social health insurance (obra social), and 5.3% had private health insurance (prepaga).

In terms of abortion method, 42.2% accessed abortion through manual vacuum aspiration (MVA), 48.0% used medication, and 6.7% used a combination of medication and MVA. The vast majority (84.6%) of survey respondents were less than 12 weeks pregnant at the time of the abortion.

#### Instrument performance

We used two indicators to assess the feasibility of the instrument: 1) the percentage of participants who completed the questionnaire, and 2) variations in item nonresponse based on certain characteristics (such as province, referring facility, and educational level). A total of 92.8% of participants completed the questionnaire in its entirety. For incomplete questionnaires, we analyzed the point at which participants stopped responding, as this could indicate a problem with the instrument. We found that eight individuals dropped out during the first section, with no discernible subsequent pattern of nonresponse.

We also examined specific questions that participants might have skipped. Only one part of the instrument had a nonresponse rate greater than 5.9%. In that case, the questions were presented in a grid format in which participants were asked to select responses within the grid. This design may have caused confusion; therefore, in the final version of the instrument, we reformulated the questions so that each one could be answered individually.

We further analyzed response variation and correlations between items to determine whether the questions measured distinct concepts and whether they correlated as expected. The questions related to quality of care included different response options depending on the item. In general, they offered three answer choices that were included in the variation and correlation analyses, plus an option for “Not sure,” which was excluded from analysis, as it was considered not to provide meaningful information.

For example, in questions about perceptions of health professionals and their treatment of patients, the options included: “Yes, at all times,” “Only at certain times / with certain people,” and “No, not at any time / with anyone.” There was also an option for “Not sure,” which was not included in the correlation analysis. Table 3 presents the percentage of respondents who answered “Yes, at all times,” or another strongly affirmative response, as a simple way to show overall trends in the data. Response variation depended on the specific item: while some questions received very high percentages of affirmative responses, others showed greater variability. Table 3 displays the items with the highest and lowest proportions of positive responses.

**Table 3:** Positive responses to questions on quality. Measuring Abortion Access and Quality (MACA) Questionnaire. Argentina, 2023-2024.

Question	Responded Yes*(%)
Did you feel comfortable expressing your needs, questions, and/or concerns to the people who attended you during the abortion process?	88.7
When you shared your personal information with those who attended you, did you feel they would maintain confidentiality?	93.4
Did you feel that those who attended you respected your decision to have an abortion?	93.9
Did you feel that those who attended you pressured you either to have or not to have an abortion?	95.8
Did those who attended you provide information about the following abortion methods: medication abortion?	98.0
Did those who attended you provide information about the following abortion methods: manual vacuum aspiration (MVA)?	74.3
Did those who attended you provide information about the following abortion methods: dilation and curettage (D&C)?	45.4
Did those who attended you talk to you about the safety of abortion methods?	97.2
Did those who attended you explain how to use the medications? [If you had a medication abortion]	99.1
Did those who attended you explain what would happen during the MVA or the D&C procedure?	93.3
Did those who attended you provide information about what you might feel during and after the abortion?	97.6
Did those who attended you provide information about warning signs to look out for?	95.3
Did those who attended you explain how to know whether the abortion was complete?	90.5
Did those who attended you explain how to manage pain during the abortion?	95.8
Did those who attended you give you the information you needed about the abortion process?	93.9
Did those who attended you ensure that you had fully understood the information provided?	95.3
Did those who attended you ask you which abortion method (medication/MVA/D&C) you preferred?	78.1
Were you able to have the abortion with the method you wanted?	94.8
Did you know what to do if you experienced a warning sign?	83.4
Were you given or recommended any medication to relieve pain during your abortion?	96.7
Did the medication you were given or recommended relieve your pain?	62.5
Did those who attended you make you feel safe during the abortion consultations?	95.7
Did those who attended you treat you with respect during the abortion process?	95.2
Did you feel supported by those who attended you during the abortion process?	91.9
Did those who attended you provide information about contraceptive methods?	98.6
Did you receive the method you wanted? [Only for those who wanted a contraceptive method; “yes” means they received it at the time of care]	81.8

Source: Own elaboration. *Answered “Yes, at all times” or another positive response. Response categories varied by question. For more information, see the published instrument.^19^

We also calculated correlations among the responses to questions related to quality of care to ensure that they followed reasonable patterns and did not reflect confusion. All items that were expected to show positive or neutral correlations did so, as did those expected to have negative or neutral correlations. No item had a correlation higher than 0.75, indicating that the questions captured distinct aspects of care, even though responses were related (Table 4). Among the three items with the strongest positive correlations, all were linked to another question: “Did the people who attended to you make sure you fully understood all the information they gave you?” This item showed strong positive correlations with the questions: “Did the people who attended to you give you the information you needed about the abortion process?”, “Did the people who attended to you give you information about what you might feel during and after the abortion?”, and “Did the people who attended to you make sure you fully understood all the information they gave you?” (Table 4).

**Table 4:** Correlations between selected questions. Measuring Abortion Access and Quality (MACA) Questionnaire. Argentina, 2023-2024.

Type of correlation	Question 1	Question 2	Correlation
Strongest positive correlations among quality questions	Did those who attended you make you feel safe during the abortion consultations?	Did those who attended you make sure that you had understood all the information they provided?	0.69
	Did those who attended you provide the information you needed about the abortion process?	Did those who attended you make sure that you had understood all the information they provided?	0.73
	Did those who attended you provide information about what you might feel during and after the abortion?	Did those who attended you make sure that you had understood all the information they provided?	0.66
Strongest negative correlations among quality questions	Were you treated with respect by those who attended you during the abortion process?	Did you feel that those who attended you pressured you either to have or not to have an abortion?	-0.26
	Did those who attended you provide the information you needed about the abortion process?	Did you feel that those who attended you pressured you either to have or not to have an abortion?	-0.26
	Did you feel supported by those who attended you during the abortion process?	Did you feel that those who attended you pressured you either to have or not to have an abortion?	-0.27

Source: Own elaboration.

After analyzing all the responses, the only questions we decided to modify were the eligibility items, in order to ensure clarity, as suggested by the healthcare professionals who invited participants to take part in the survey.

As a complementary tool - and with the aim of facilitating the transfer and application of the survey - we developed a patient manual^20^ and designed the instrument in Google Forms so it could be accessed and implemented by anyone interested in applying the survey in a healthcare setting (see supplementary materials).

## DISCUSSION AND CONCLUSIONS

Law 27,610 was the result of a long history of activism, coalition-building, and legislative advocacy.^2^^,^^21^ As such, the Voluntary Termination of Pregnancy (VTP) law had a high public profile and was backed by widespread social mobilization and broad political support, while also facing strong ideological opposition making the monitoring of its implementation as a public policy essential.

The World Health Organization (WHO) defines monitoring as the process of repeatedly observing a situation to detect changes over time.^22^ Measuring access to and quality of care in Argentina constitutes a form of monitoring focused on the microsocial level of health services. As is well known, quality care must be centered on the patients, responsive to their expectations, and aligned with the fulfillment of their right to health.^23^

Understanding patients’ experiences with abortion services allows for the evaluation of healthcare teams’ performance, the identification of areas for improvement, and the provision of information to decision-makers about the barriers people face when navigating the system. Additionally, these data can help identify inequalities across different regions and among populations with diverse characteristics and needs. With these goals in mind, we developed and validated an instrument to measure accessibility and quality of abortion services in Argentina.

In developing the instrument, our primary objective was to achieve content validity. Once we identified international standards for measuring quality of care, these needed to be translated, adapted, and validated within our specific context. We conducted our study to incorporate and assess expert validity, relying on experienced professionals familiar with the healthcare system and patient populations across regions of the country.^18^

Our study’s strengths include consulting a wide variety of stakeholders in the field to ensure the theoretical and technical validity of the MACA questionnaire as a relevant instrument for the Argentine context and one that could be applied by other research teams. Secondly, the pilot test evaluated the instrument and its indicators across diverse regions and healthcare services, providing evidence of its feasibility and reliability. As a result, the MACA questionnaire includes indicators representing various types of service accessibility (symbolic, organizational, geographic, and economic), as well as multiple domains of care quality (interaction between patient and provider, information provision, decision-making, and technical competence).

The MACA questionnaire was designed to be applied across all three subsectors of Argentina’s healthcare system. In this regard, it differs from the ACQTool, which was developed to measure quality in community services, pharmacies, and telephone hotlines as well.^15^ These contextual differences and the specific purpose of monitoring the recent implementation of Argentina’s abortion access policy led to the selection of different indicators than those included in the ACQTool. Thus, we incorporated indicators of symbolic accessibility (such as patients’ knowledge of abortion legality and where to request services), indicators on access pathways (number of visits, number of services), and geographic accessibility (travel time), along with subjective indicators to assess expectations of care (perceived acceptability of wait times and number of visits required to receive care).

From an intersectional perspective, we deliberately included questions from the outset about gender identity, disability, Indigenous or Afro-descendant identity, migration status, place of residence (whether in the capital city or the interior of a province), education level, age, and occupation. These dimensions allow us to describe how accessibility and quality of care vary across multiple axes of social inequality, which intersect and co-produce conditions of greater or lesser vulnerability.

At the same time, we took into account local practices and the standards established by law. In our context, it is relevant to record the number of days between the request for an abortion and its completion in order to assess adherence to the regulation, which establishes a maximum period of 10 days. Similarly, we replaced a subjective indicator - such as the perceived affordability of services for patients - with a more objective measure: any out-of-pocket expenses incurred to access VTP/LTP procedures, in order to assess compliance with the law mandating comprehensive and free coverage of abortion services.^21^ Recognizing that the eradication of curettage as an abortion procedure remains an unresolved issue in the country - despite it being explicitly discouraged by the WHO and the national protocol - we decided to inquire separately about each abortion method and identify where curettage is still being used, with the aim of potentially promoting training in appropriate techniques.^3^^,^^28^^,^^29^

The open-access publication of the *Manual for the Implementation of the MACA Survey,*^20^ as well as the publication of the questionnaire as supplementary material to this article, aims to enable its use in diverse settings. Among the limitations of this study, it is worth noting that the pilot test was conducted in six provinces, although the MACA Survey was designed as a nationally applicable monitoring tool. Access to the target population - namely, patients accessing abortion services - was facilitated through healthcare professionals who invited them to participate. We acknowledge that this may have introduced a courtesy bias, with patients potentially providing more favorable responses toward those who cared for them, even though the questionnaire was anonymous and self-administered by patients on their own devices, at a time and place of their choosing.

Although quality of care was broadly defined and multiple dimensions were considered, not all are included in the instrument. The MACA questionnaire does not gather data on health services’ referral systems or supply chain management. It also does not evaluate clinical outcomes (such as effectiveness, complication rates, or other health events), nor does it assess the perspectives of healthcare professionals or their potential emotional or workload-related stress, among other aspects. Furthermore, the MACA questionnaire captures the quality of care only in relation to the staff who provided abortion services and invited patients to participate. As such, it excludes reception or administrative staff and personnel from other departments (e.g., ultrasound, referrals), as well as those involved in care denials or inadequate interactions. Therefore, unless it is part of a centrally coordinated public intervention to improve abortion quality, it is likely that the questionnaire will be applied by individuals and institutions already committed to high-quality care, and that the results observed will reflect better performance in these settings compared to the broader landscape of sexual and reproductive health services in the country.

Nonetheless, based on the key topics identified by the panels of experts, the MACA questionnaire aligns closely with international standards and reflects the four dimensions of quality that have been validated in multiple global contexts.^6^ The emphasis placed on indicators related to emotional support and accompaniment is consistent with other research conducted in Argentina, which highlights the value patients place on long-term support, trust, and guidance throughout the abortion process.^10^^,^^28^^,^^30^ This combination of internationally recognized dimensions and a contextually adapted approach ensures that the instrument adequately measures key aspects of accessibility and quality. Finally, the MACA questionnaire is proposed as a tool for monitoring the implementation of the sexual and (non-)reproductive rights guaranteed by Argentine law. We hope it will be useful for activists, healthcare teams, and public policy decision-makers in advancing the implementation of abortion policy in Argentina and improving patient satisfaction with the healthcare system.

## SUPPLEMENTARY MATERIAL

Questionnaire designed in Google Forms for the MACA Survey (Medimos Acceso y Calidad del Aborto) is available online (see Supplementary Material).
